# Endosperm cell size reduction caused by osmotic adjustment during nighttime warming in rice

**DOI:** 10.1038/s41598-021-83870-1

**Published:** 2021-02-24

**Authors:** Hiroshi Wada, Fang-Yu Chang, Yuto Hatakeyama, Rosa Erra-Balsells, Takuya Araki, Hiroshi Nakano, Hiroshi Nonami

**Affiliations:** 1 Kyushu Okinawa Agricultural Research Center, National Agriculture and Food Research Organization, Chikugo, Fukuoka Japan; 2 Graduate School of Agriculture, Ehime University, Matsuyama, Ehime Japan; 3 The United Graduate School of Agricultural Sciences, Ehime University, Matsuyama, Ehime Japan; 4 Kaohsiung District Agricultural Research and Extension Station, Council of Agriculture, Executive Yuan, Pingtung, Taiwan; 5 Department of Organic Chemistry and CIHIDECAR (CONICET), University of Buenos Aires, Buenos Aires, Argentina

**Keywords:** Analytical chemistry, Plant stress responses, Heat

## Abstract

High night temperature (HNT) often reduces yield in field crops. In rice, HNT during the ripening stage diminishes endosperm cell size, resulting in a considerable reduction in final kernel weight; however, little is known about the underlying mechanisms at cell level. In this study, we performed picolitre pressure-probe-electrospray-ionization mass spectrometry to directly determine metabolites in growing inner endosperm cells of intact seeds produced under HNT conditions, combining with ^13^C feeding and water status measurements including in situ turgor assay. Microscopic observation in the inner zone suggested that approximately 24.2% of decrease in cell expansion rate occurred under HNT at early ripening stage, leading to a reduction in cell volume. It has been shown that HNT-treated plants were subjected to mild shoot water deficit at night and endosperm cell turgor was sustained by a decline in osmotic potential. Cell metabolomics also suggests that active solute accumulation was caused by a partial inhibition of wall and starch biosynthesis under HNT conditions. Because metabolites were detected in the single cells, it is concluded that a partial arrest of cell expansion observed in the inner endosperms was caused by osmotic adjustment at mild water deficit during HNT conditions.

## Introduction

High temperature during grain filling stage has been widely taking a toll on the production of field crops, which induces a reduction in grain growth, resulting in yield loss^1, 2^. Most studies have focused on high day temperature-related responses; however, information on HNT responses is still limited particularly for the understanding of cell-specific events. In rice, it is known that HNT affects its physiology in all stages from vegetative through ripening stages, leading to various negative impacts on rice yield^2, 3^. Long-term HNT conditions (i.e., the 5th day after heading (DAH) until maturation) during the ripening stage severely affect grain development throughout a reduction in endosperm cell size and decline grain weight^4^. Based on the fact that the partial heat exposure of the leaves and culms except for panicles induced no reduction in grain weight, it has been pointed that a reduction in grain weight at HNT may not be directly caused by the deficit of carbohydrates in the vegetative parts due to increased respiration loss^5^. Although another factor(s) might influence the endosperm growth at HNT, what causes such a reduction in cell volume during HNT conditions remains unknown.

More recently, it has been pointed out the impact of nighttime atmospheric vapor pressure deficit (VPD) on changes in rice physiology under HNT, rather than HNT itself^6^. It should not be ignored that increasing VPD reduces air water potential, causing temporal shoot water deficit^7, 8^. Dry wind conditions during middle ripening stage also induce temporal shoot water deficit to produce ring-shaped chalky kernels throughout osmotic adjustment with no reduction in kernel weight if the duration is relatively short (i.e., < 24 h)^8^. The reduction in kernel weight has been recorded when the duration extends > 2 days^9^. In rice endosperms, cell expansion occurs first, followed by active starch accumulation at the early ripening stage^10, 11^. Starch accumulates from the innermost cells towards the cells of the peripheral part of endosperms^12^. Water uptake and wall expansion both need to occur during cell expansion. Therefore, if HNT conditions similarly induce shoot water deficit at night, it is expected that plant water status may be disturbed to diminish the cell size before starch granules have been packed. However, there have been no direct analysis conducted on the responses from the viewpoint of plant-water relations. In addition, little is known about changes in metabolisms during cell expansion in the endosperms growing during nighttime.

Electrospray Ionization (ESI) is a widely used mass spectrometry ionization chamber that allows a straight analysis of analytes in polar solution through the coupling of LC/MS or direct infusion (DI-ESI) analysis. A cell pressure-probe^13^ long-used to directly determine turgor pressure in plant cells, has been combined with an Orbitrap mass spectrometer to establish as in situ cell metabolomics, termed “Picoliter pressure-probe electrospray-ionization mass spectrometry (picoPPESI-MS)” (previously named: internal electrode capillary (iec) electrospray-ionization mass spectrometry, IEC-PPESI-MS^14^). In this method, sample solution is infused in the chamber through a specially sealed needle-electrode embedded in the quarz-capillary attached to the housing of pressure-probe (see Fig. S2 in Ref.^14^), which yields poly-charged macro drops. Because of the action of a very high electric field on this poly-charged drop of the solution, the desorption/desolvation/volatilization of the analytes take place and intact analyte molecules as gas-ions are formed (soft ionization method). These gas-ions are focused to the Orbitrap mass spectrometer. This analytical method allows to collect information about (1) analyte molecular weight and (2) the chemical structure of the analyte by inducing the fragmentation/decomposition of the selected intact molecule gas-ion (precursor ion) (MS/MS experiments).

In this study, we have hypothesized that the inner endosperm cells might be osmotically adjusted at moderately low water potential, leading to a partial inhibition of cell expansion under nighttime warming conditions. To test this hypothesis, we have combined cell metabolomics with ^13^C feeding and water status measurements. For cell metabolomics, we have utilized picoPPESI-MS to directly assay cell metabolites and turgor pressure in growing single endosperm cells of intact seeds produced under HNT conditions environmentally controlled in on-site cell-specific analytical method described previously^15–18^. Here, we show that HNT conditions imposed the plants to mild water deficit at night and the expanding inner cells were adjusted osmotically, causing several metabolic changes, reducing cell size, resulting in a decline in final kernel weight.

## Results

### Final kernel weight and cell size

**Figure 1:**
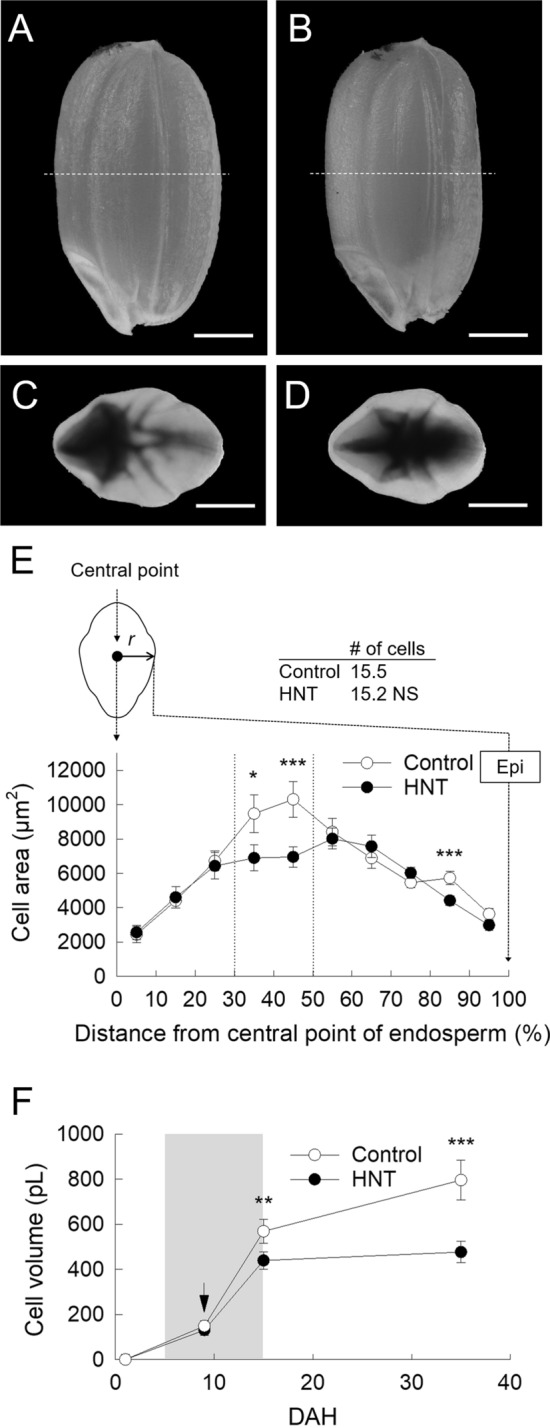
Images of mature rice kernels in control (**A**, **C**) and HNT treatment (**B, D**). (**C**) and (**D**) indicate the top view of the mature kernels. 35 DAH endosperm cell area plotted against the distance (in %) from central point to the lateral epidermis of transverse sections (corresponding to the dashed line in **A** and **B**) of the kernels in each treatment (**E**). In the inset in (**E**), number of endosperm cells across the same zone was counted. Epi, epidermis; NS, no significance. Time course of changes in inner endosperm cell volume (corresponding to the dotted line in **E**, 30–50% distance) in each treatment (**F**). White and black circles in (**E**) and (**F**) indicate the control and HN treatments, respectively. The gray area in (**F**) indicates the duration of treatment; an arrow indicates the time conducting picoPPESI-MS and water status measurements. Data in (**E**) are the mean ± SE of 7–64 individual cells collected in 4–5 kernels from three plants. Data in (**F**) are the mean ± SE of 30–58 individual cells collected in 3–5 kernels from three plants.*, ** and *** shows *p* < 0.1, 0.05 and 0.01 by *t-*test, respectively. (**A**–**D**) scale bars = 1 mm.

The rice plants were exposed to the 10 day HNT conditions and grain weight, grain dimension, and cell anatomy were measured at maturation. A substantial reduction (ca. 5.7%) in final kernel weight has been observed in HNT-treated kernels (Table 1). Although kernel length thickness was sustained under HNT conditions (Table 1, Fig. 1A, B), kernel width and grain volume both declined under HNT conditions, resulting in a remarkable decline in final kernel weight (Table 1, Fig. 1C, D). The image analysis of inner endosperm cells of 35 DAH kernels, around 30–50% distance from central point in lateral side, where a cell pressure probe was introduced, shows after treatment difference in the cell size mainly caused by the inhibition of cell expansion at HNT (Fig. 1E) with a partial occurrence of basal-white and white-back rice (Fig. 1B) and number of cells in both treatments were similar in the endosperms (Fig. 1E). Time course of cell volume in above-mentioned region shows that there is no difference between treatments at 9 DAH, but at the end of stress treatment, 15 DAH, a significant decreasing in cell volume of HNT treatment was observed (Fig. 1F). Additionally, we also observed that central point of endosperms moved towards dorsal side under HNT conditions (Fig. S1).

**Table1:** Grain weight and dimension (grain length, width, thickness, and volume) in control and HNT treatment at maturation.

Treatment	Grain weight (mg)	Grain dimension			
		Grain length (mm)	Grain width (mm)	Grain thickness (mm)	Grain volume (mm^3^)
Control	21.27	4.99	2.90	2.06	15.63
HNT	20.07	4.97	2.79	2.05	14.87
Treatment effect	*	NS	***	NS	**

Data of grain weight are the mean ± SE of 5–6 individual plants, 8 grains per plant.

*, ** and *** shows *p* < 0.05, 0.01 and 0.001 by Student’s *t*-test, respectively. *NS,* no significant difference (Student’s *t*-test, *p* > 0.05). For the determination of grain dimension, see “Materials and Methods” section.

### ^13^C distribution percentage

**Figure 2:**
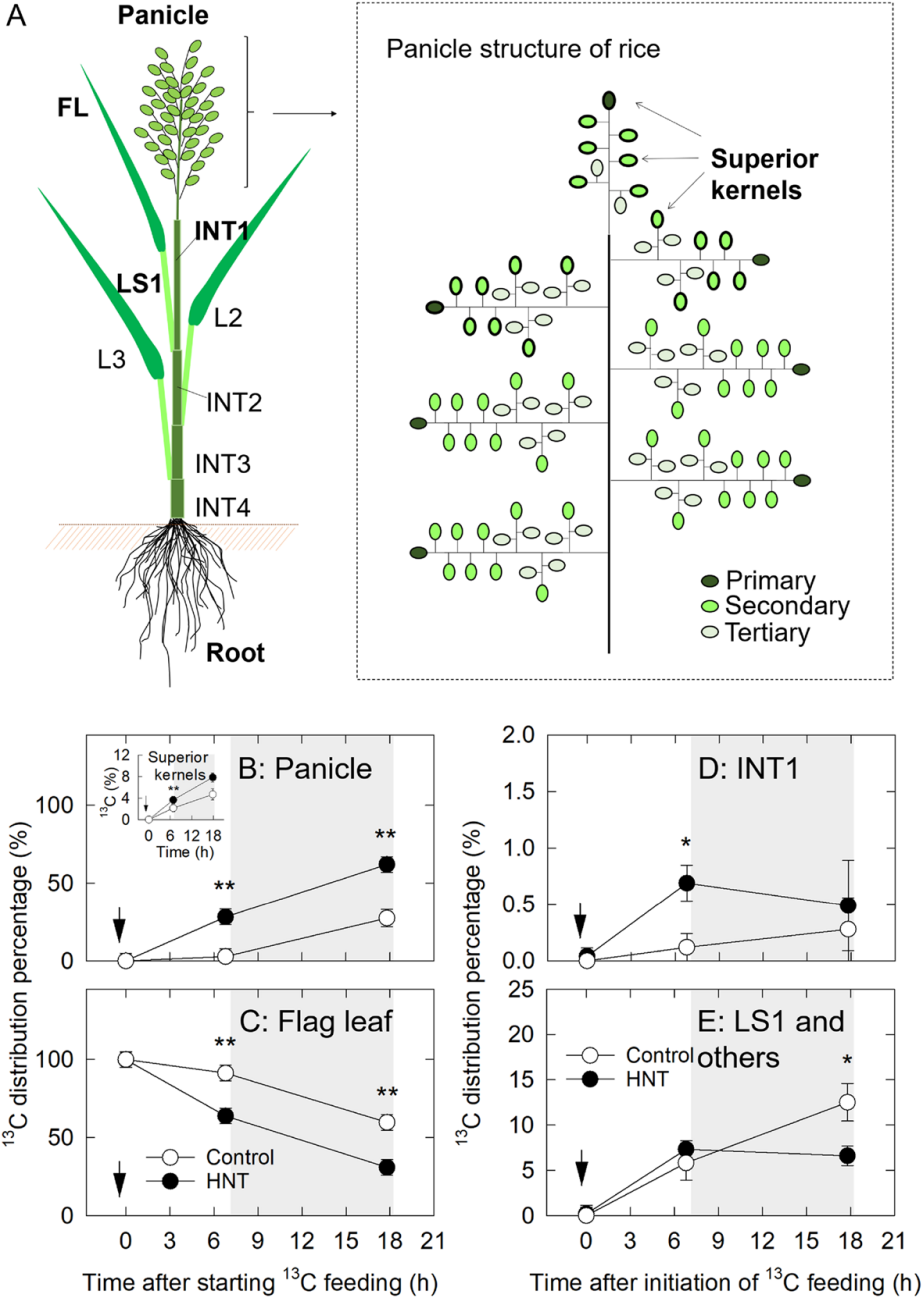
(**A**) Diagram of rice plant (left) and rice panicle structure (right). Time course of changes in ^13^C distribution in the panicle (**B**); in the flag leaf (**C**); in the INT1 (**D**) and in flag leaf sheath and others (determined as pooled organs with these tissues) (**E**). In panicle structure in (**A**), superior kernels attached to the same position, where the on-site cell metabolomics was conducted, were displayed with thick lines. Changes in ^13^C distribution in the superior kernels were also shown (see inset in **B**). The plants were labeled with ^13^CO_2_ for 30 min from the flag leaf at 8 DAH at day time, as indicated by the arrow in (**B**–**E**). White and black circles indicate the control and HNT treatments, respectively. Gray areas indicate the HNT treatment. *FL,* flag leaf; *INT1*, first internode; *LS1,* first leaf sheath; *L2*, second leaf blade. Each point is the mean ± SE of four samples from different plants. * and ** show *p* < 0.05 and 0.01 by *t*-test, respectively.

To examine the effect of HNT treatment on ^13^C distribution in the plants, plants were subjected to HNT conditions after ^13^C was pre-fixed. There was no detectable ^13^C distribution in roots during the treatment duration, and ^13^C distribution in other tissues (panicle, superior kernels, flag leaf, the first internode (INT1) and the first leaf sheath (LS1)) was shown in Fig. 2. Just after ^13^C was fed in flag leaf at 8 DAH, ^13^C mass per flag leaf dry weight in HNT treatment and control was 3.11 ± 0.26 mg g DW^−1^ (n = 4) and 3.02 ± 0.13 mg g DW^−1^ (n = 4), respectively. This suggests that treatment difference in ^13^C partitioning was simply related to translocation capability under different water status. According to the decline in ^13^C distribution percentage in flag leaf, ^13^C entry in HNT-treated panicle remarkably increased in the daytime set even at normal day temperature (Fig. 2B, C). In panicle, ^13^C distribution percentage in HNT-treated superior kernels, where we conducted cell metabolomics (see below), dramatically increased toward the next day, compared with control (Fig. 2B inset). During daytime in HNT treatment, an rapid increase in ^13^C distribution in the culms and sheaths was observed, whereas ^13^C distribution in control was shown to gradually increase through the night (see the first internode, first leaf sheath, and others, Fig. 2D, E).

### Water status in panicle and endosperm at nighttime warming

Predawn panicle water potential in HNT treatment declined down to – 0.46 MPa, whereas that of control remained – 0.30 MPa (Table 2). In the expanding stage of score 0.8 kernels, the tissue-averaged kernel water potential was highly correlated with panicle water potential (PWP), consistent with the previous works^8, 9^. The regression line between PWP (*x*) and kernel water potentials (*y*) was *y* = 0.91*x*– 0.07 with *r*^2^ = 0.92 (*p* < 0.001) (Fig. S2), and the relationship was linear and essentially equivalent across a broad range of PWP in two treatments, ranging from – 0.27 to – 0.58 MPa. There was no treatment effect on cell turgor (Table 2). The kernel water potential, calculated from the above-mentioned regression line, was reasonably assumed to be equilibrated with the water potential of expanding cells during the measurement with isopiestic psychrometers^8^. When the osmotic potential (negative quantity of osmotic pressure) of endosperm cells was calculated by subtracting cell turgor from kernel water potential, the osmotic potential under HNT treatment was shown to be lower than that of control (Table 2). The growth-induced water potential in control and HNT treatment calculated as the difference of water potential between xylem water potential (i.e., PWP) and expanding cells was 0.04 MPa and 0.03 MPa, respectively, with a significant treatment difference (Table 2).

**Table 2:** Panicle water potential, kernel water potential, endosperm cell turgor, calculated osmotic potential, and growth-induced water potential determined at 8 DAH nighttime in control and HNT treatment.

Treatment	Panicle water potential (MPa)	Kernel water potential (MPa)	Turgor (MPa)	Calculated osmotic potential (MPa)	Growth-induced water potential (MPa)
Control	− 0.30	− 0.34	0.01	− 0.35	0.04
HNT	− 0.46	− 0.49	0.01	− 0.50	0.03
Treatment effect	***	***	NS	***	***

Kernel water potential was calculated by regression equation (see “Results” section). Cell turgor was directly determined at 9 DAH. The calculated osmotic potential was calculated by subtracting turgor from kernel water potential. The growth-induced water potential was calculated by subtracting the kernel water potential from panicle water potential (source of water). The data of panicle and kernel water potential are means ± SE from 6 to 9 independent plants in each treatment. The data of turgor are means ± SE from 12 to 15 cells from 5 to 8 kernels from three independent plants in each treatment

*** and NS indicate *p* < 0.001 (*t*-test) and no significant difference, respectively.

### Metabolomic changes at nighttime

**Figure 3:**
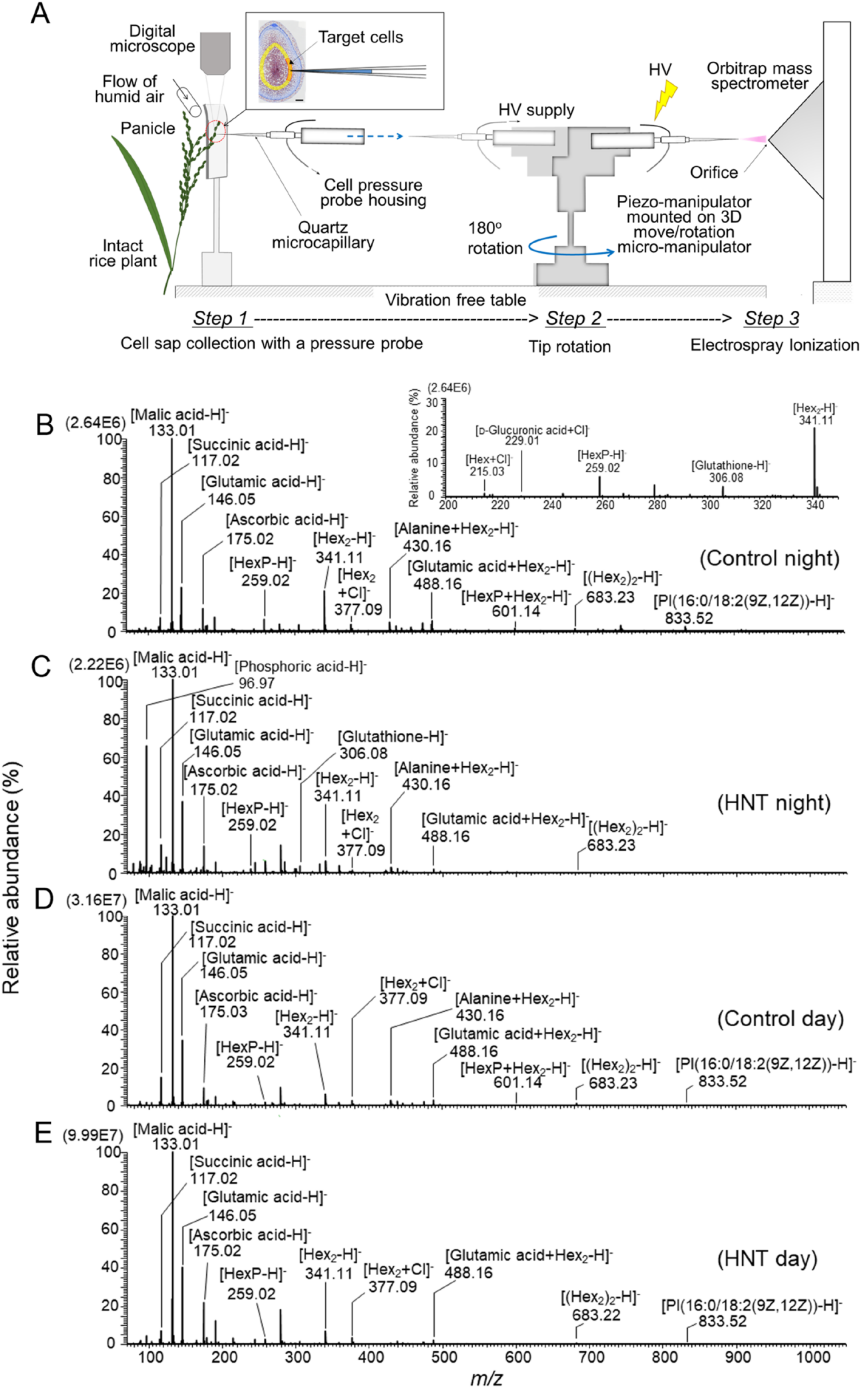
(**A**) Schematic diagram showing the flow from on-site cell metabolomics conducted in the rice endosperm cells growing under controlled environments. After cell sap collection using a cell pressure probe (Step 1), the tip was immediately rotated (Step 2) and then picoPPESI-MS was performed under the same environements (Step 3) (also see “Methods” section). Scale bar in the transverse section embedded in **A** is 200 μm. (**B–E**) Negative ion picoPPESI mass spectra obtained from the cells in control and HNT treatment at 9 DAH daytime and nighttime. Inset figure in **B** indicates magnified range of *m/z* 200–350 to show peaks of [Hex + Cl]^−^, [d-Glucuronic acid + Cl]^−^, [HexP-H]^−^, [Glutathione-H]^−^, and [Hex_2_-H]^−^. The data are representative of repeated experiments with 9–14 kernels in each treatment.

**Figure 4:**
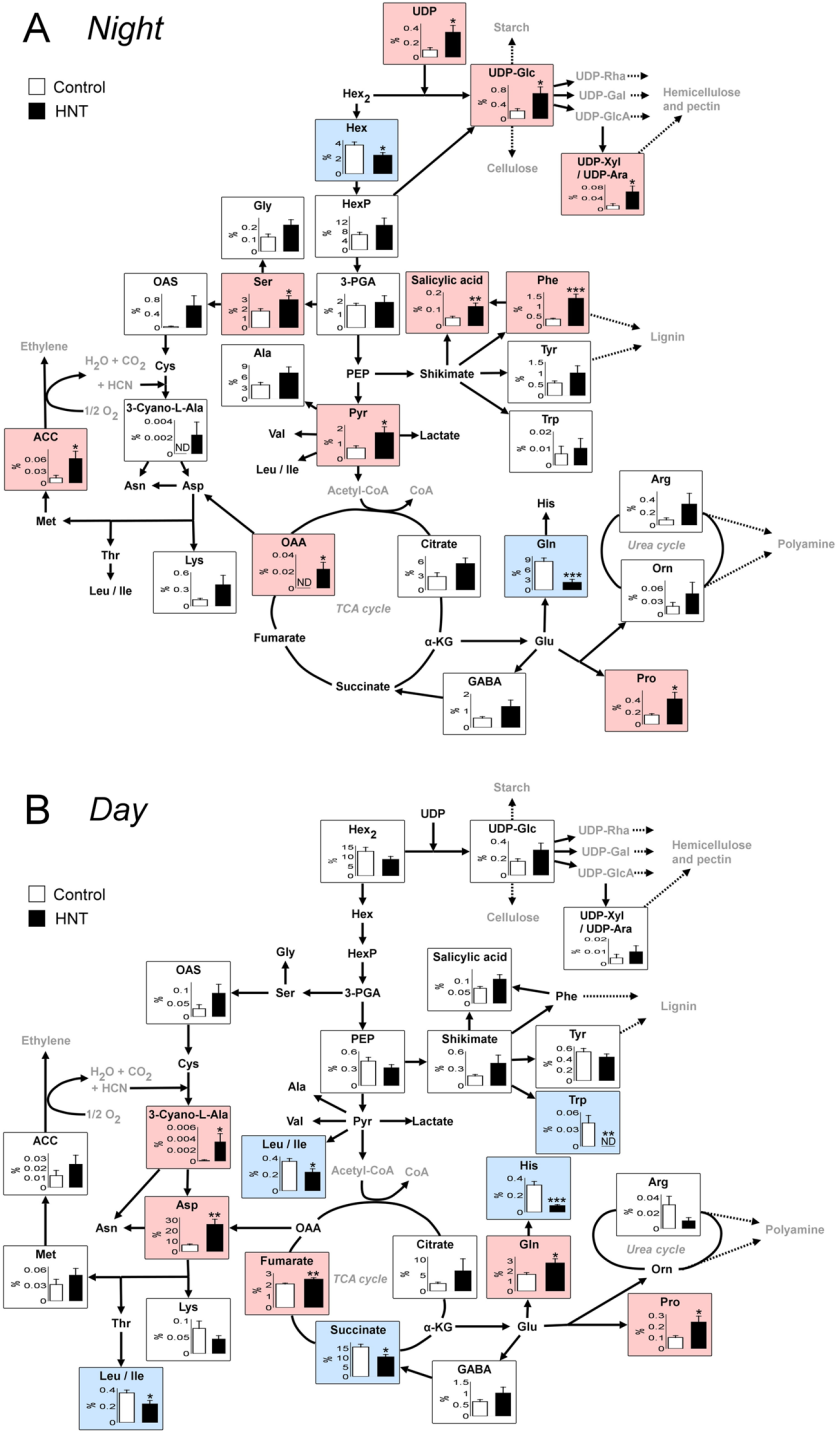
Changes in cell metabolisms in the 9 DAH inner endosperm cells at nighttime (**A**) and daytime (**B**) under HNT conditions. White and black bars indicate control and HNT treatment, respectively. Metabolites highlighted in pink and blue indicate that the relative abundances are significantly higher and lower under HNT treatment, respectively. The data are means ± SE from 9 to 14 kernels from at least three independent plants in each treatment. *, ** and *** show *p* < 0.05, 0.01 and 0.001 by *t-*test, respectively.

When in situ single-cell metabolomics at real time in negative ion mode was performed in the expanding inner endosperm at night, abundant metabolite signals were identified (Fig. 3, Table S1, Fig. S2–S5). In control, the peaks of malic acid, glutamic acid, Hex_2_, succinic acid and ascorbic acid as [M − H]^−^, (M = molecular mass) were observed as the top five higher intensity signals (Table S1). A similar pattern has been observed in HNT treatment; the peaks of deprotonated malic acid, glutamic acid, phosphoric acid, ascorbic acid, and Hex_2_, were the top five major signals (Table S1). Although signal intensity value of sugars (except for pentose) as [M + Cl]^−^ in HNT treatment was slightly smaller than control, observed signal intensity of the majority of amino acids (except for glutamine) in the same treatment was higher than in control, at night (Fig. 4A and Table S1). In particular, the content of proline, phenylalanine, and serine, was significantly higher than control (Table S1). Importantly, the content of cell wall realated metabolites, such as *p*-coumaric acid, UDP-glucose, UDP-d-xylose (arabinose) and UDP; redox related metabolites, such as monodehydroascorbic acid, dehydroascorbic acid and glutathione; stress response plant hormones, such as salicylic acid and 1-aminocyclopropane-1-carboxylic acid (ACC) (the known precursor of ethylene); one of metabolic branch points, pyruvic acid, in HNT treatment were greater than control (Table S1). On the contrary, the content of phosphatidylinositol (PI(16:0/18:2(9Z,12Z))), dramatically declined under HNT conditions (Fig. 3B, C, Table S1, and Figs. S5 and S6). Besides, there was no change for cysteine signals (as [cysteine-H]^−^ and the cluster [cysteine + Hex_2_-H]^−^) between treatments and the intensity of the cluster [cysteine + Hex-H]^−^ signal slightly diminished under HNT conditions.

### Metabolic changes at daytime

In contrast to the various nighttime responses, there were less treatment differences in metabolic changes and less accumulation of metabolites in HNT treatment during daytime (Table S1). Importantly, among metabolites related to cell wall synthesis, which were observed significantly different during nighttime, only α-l-rhamnose was higher under HNT treatment during daytime (Fig. 4 and Table S1). Overall, the content of sugars and amino acids was slightly lower in daytime, compared with nighttime. It is notable that the relative abundance of proline was still higher under HNT treatment at daytime; glutamine showed an opposite response between daytime and nighttime (Fig. 4). During the daytime, HNT-treated cells also exhibited greater content of ascorbic acid, aspartic acid, fumaric acid, glycerol, and 3-cyano-l-alanine that participates in detoxification of cyanide, compared with control. The same behavour was not observed for succinic acid, histidine, tryptophan, leucine or isoleucine as [M − H]^-^, and some sugar cluster ions, [Alanine + Hex_2_ − H]^−^ and [Succinic acid + Hex_2_ − H]^−^ (Table S1).

## Discussion

Microscopic observation shows that a partial inhibition of cell expansion occurred in the inner endosperms under 10 day HNT conditions (Fig. 1), resulting in approximately 5.7% of reduction in final kernel weight (Table 1). Unlike foehn-induced dry wind conditions (PWP = ca. – 0.90 MPa)^8^, predawn PWP at HNT was – 0.46 MPa, indicating that nighttime shoot water deficit was unexpectedly mild. Similar to other rice studies reported in leaves^19–21^ and endosperms^8^, an decrease in osmotic potential sustaining turgor occurred in the inner endosperm cells in HNT treatment (Table 2), indicating that osmotic adjustment occurred at least in the cells at nighttime warming. ^13^C feeding analysis shows that at least between 8 and 9 DAH (Fig. 2), which corresponds to the timing of maximum rate of increasing grain dry weight in HNT treatment^4^, sink activity was maintained. On-site cell metabolomics performed under HNT conditions discriminated treatment differences in metabolites mainly associated with wall and starch biosyntheses and ascorbate–glutathione pathway, together with sugar and amino acid accumulation (Figs. 3, 4 and Table S1). These data strongly suggest that solutes accumulated into the cells would have originated from materials to be used for cell expansion and starch accumulation, resulting in the size reduction in the inner endosperm cells scavenging ROS to cope with nighttime warming. Taken all together, we conclude that the endosperm cell size reduction locally observed under HNT conditions was caused by osmotic adjustment (Fig. 1, Table 2).

### Impact of shoot water deficit under nighttime warming

In the present study, we have investigated physiological causes on cell size reduction during early ripening stage caused by HNT conditions in the environmentally controlled chambers, in order to cancel out potential candidates including heat history prior to ripening. In addition, to rule out the potential effects from other yield-related components (e.g., the number of tillers) that affects kernel weight, we only kept main stem per plant in this study. Consistent with the previous reports^7, 8^, an increase in VPD dramatically reduces air water potential, and consequently well-watered plants were subjected to temporal shoot water deficit, as indicated in Table 2. Daytime environmental conditions in two treatments of this study were same (see “Methods” section, 28.0 °C 70% RH [i.e., VPD = 11.3 hPa]). However, at night, VPD decreased down to 5.3 hPa in control, whereas that of HNT treatment was 10.6 hPa, corresponding to – 31.6 MPa of air water potential. Because PWP (i.e., xylem water potential in panicle) in HNT was – 0.46 MPa (Table 2), the water potential gradient established between air and panicle in HNT should be 31.2 MPa, much smaller than that of 24 h foehn-induced dry wind conditions (125.6 MPa)^8^. Therefore, the extent of nighttime water deficit in this study was suggested to be surprisingly mild. Given the fact that carbon transport actively occurred to the growing kernels during daytime in HNT treatment (Fig. 2A) and less accumulation of solutes at daytime (Fig. 4B), shoot water deficit would be released during daytime, causing a reversible effect on rice physiology.

### Regulation of endosperm cell expansion under HNT conditions

Morita et al.^4^ applied same HNT treatment (34 °C, 80% RH at night) throughout ripening stage (for 40 days) and observed severe cell volume reduction over a wide range of endosperms with shorter duration of grain growth than control. When conducted 10 day HNT treatment in this study, inhibition of cell expansion was confined within a part of inner endosperms (see Fig. 1). The main difference in endosperm cell morphology between two studies was attributed to the duration difference, rather than difference in intensity of water deficit. Under HNT conditions, an inhibition of cell expansion mainly occurred from dorsal to lateral endosperms and a reduction in grain width (Fig. 1 and Table 1), also leading to a remarkable shift of the central point towards the dorsal epidermis (Fig. S1), consistent with Nagato and Ebata^22^. Regarding the grain growth duration, the maximum grain growth rate between HNT treatment and control was suggested to be 13 DAH and 16 DAH, respectively^4^. In this study, we have used the grains with the same developmental stage to study cell expansion process at 9 DAH, so that the potential age difference between treatments could be minimized. It has been reported that a decrease in grain dry weight observed under HNT conditions was mainly caused by a reduction in cell expansion rate^4^. When plant cell expands, the water potential gradient associated with growth (i.e., growth-induced water potential) would be established between xylem and expanding cells^23, 24^, even when transpiration occurs simultanelously^25^. The data suggest that a reduction in growth-induced water potential might occur at HNT (Table 2). Additionally, cell turgor is required to expand wall during cell expansion; however, there was no treatment difference in turgor (Table 2). Although cell hydraulic conductivity and wall extensibility associated with cell expansion rate through metabolic alterations have not been determined in this study, these parameters might also be associated with the regulation of cell expansion at HNT, as discussed below.

### Metabolic changes at osmotic adjustment under HNT conditions

Combining water status measurements with in situ intact cell metabolomics indicate that cells were adjusted osmotically at HNT, similar to those observed under dry wind conditions^8, 9^. Also, turgor maintenance was observed by solute accumulation, mainly sugars and amino acids, similar to the early study^26^. An increase in osmotic pressure was 0.15 MPa (see Table 2), corresponding to approximately 61.5 mM (when calculated as sucrose). Metabolic changes in sugars and amino acids may refer to the slowdown in metabolic rate, presumably to minimize energy consumption at osmotic adjustment, increasing osmotic pressure to partially sustain cell expansion through turgor maintenance (Table 2). Other main solutes detected, such as wall-related compounds and redox metabolites in intact cells would also take part in the increase in osmotic pressure (Fig. 4A, Table S1 and Table 2). Henzler and Steudle^27^ treated the alga, *Chara corallina* with hydrogen peroxide to observe a reduction in cell hydraulic conductivity, through the deactivation of channel, which shows a close relationship between membrane permeability and redox status^28^. If similar metabolic alteration had occurred in the membrane under HNT conditions, this may partially explain the observed reduction in cell size, as addressed above.

**Figure 5:**
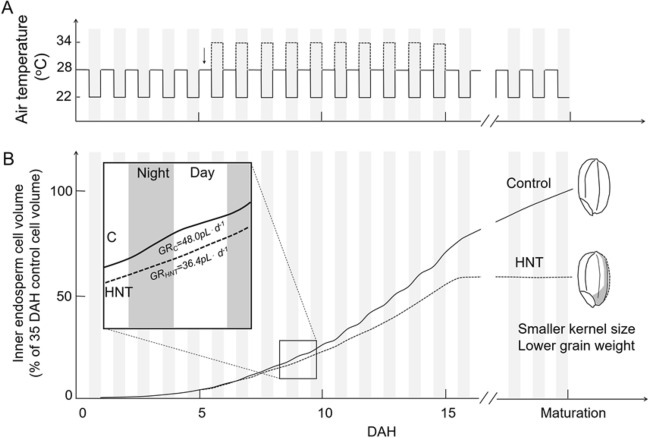
Air temperature setting of 10 day HNT treatment conducted during early ripening stage in this study (**A**). Arrow in (**A**) indicates initiation of HNT treatment. Putative changes in cell volume in inner endosperms under HNT conditions throughout development (**B**) (see Fig. 1). The graph was generated based on the cell volume data shown in Fig. 1F, assuming that cell growth rate during daytime was similar to control. Inset in (**B**) shows expanded putative diurnal growth curve, and the cell growth rate (*GR*) in control and HNT was estimated to be 48.0 pL . d^−1^ and 36.4 pL . d^−1^, respectively. Solid and dashed lines in (**A**) and (**B**) indicate control and HNT treatment, respectively.

Similar to high day temperature (HDT) conditions^16^, an increase in content of redox-related metabolites, such as dehydroascorbic acid, monodehodroascorbic acid, and glutathione at night and ascorbic acid at daytime was observed, at the stage examined (Table S1). In the endosperms treated at HDT, considerable accumulation of cysteine that forms disulfide bridges has been observed prior to the chalky formation throughout a reduction in cytosolic protein synthesis^16^. In contrast to HDT, it has been shown that there were little changes in cysteine content between two timings in the inner endosperms under HNT conditions (Table S1). This suggests that cysteine might not participate in osmotic adjustment and protein synthesis itself might not be considerably inhibited by HNT, contrastingly different from the responses to HDT conditions^16^. ^13^C analysis suggests that starch biosynthesis might be sustained by adequate assimilate supply at low water potential (Fig. 2). Consequently, starch granules (and protein bodies) are most likely to completely fill up the cells whose volume was partially reduced throughout osmotic adjustment, as no chalkiness was observed in lateral side (Fig. 1B). Furthermore, the cell metabolomics described here illustrates that several key metabolic pathways associated with cell expansion are likely to be retarded during HNT conditions, as can be seen in Fig. 5 (75.8% of control at 9 DAH). UDP-glucose is the precursor of cellulose synthesis. Materials for matrix polysaccharides, such as UDP-glucose and pentose appeared to remain in cytosol without supplying from cytoplasm to the wall, causing a slight decline in cell expansion rate in the inner zone. In *Arabidopsis*, the delivery of cellulose synthase (CESA) complex to plasma membrane was inhibited under osmotic stress^29^. Here, ^13^C analysis combined with water status measurement shows greater carbon supply from source to sink occurred at shoot water deficit (Fig. 2 and Table 2), although greater accumulation of *p*-coumaric acid, UDP-glucose, UDP-d-xylose (arabinose), and UDP simultaneously occurred in the target cells (Fig. 4A and Table S1); this fact strongly suggests a partial inhibition of wall biosynthesis caused by osmotic adjustment under HNT conditions. Although cell wall would be remodeled by pectin methylesterase under heat stress^30^, our data showed that there might be less effect on pectin-related metabolites at nighttime, except for α-l-rhamnose (*p* = 0.09) (Table S1). As consequence, these metabolic changes might alter wall extensibility, as mentioned above. Although there was no statistical treatment difference in cell volume at 9 DAH (Fig. 1F), ^13^C feeding experiment (8–9 DAH), water status measurement, and metabolites changes at 9 DAH reveals clear treatment differences in water relations and metabolisms in the inner cells during nighttime. The opposite pattern of diurnal changes in glutamine content may reflect to phloem unloading (Fig. 4, Table S1).

It is known that cell wall synthesis and cell expansion could be asynchronous, and typically cell expansion occurred faster at nighttime due to the higher water potential at low evaporative demand^31^. Therefore, we gave a putative growth of inner endosperm cell volume (Fig. 5) in this study. In control treatment, the growth rate is faster at nighttime; in HNT treatment, because of the mild water deficit and osmotic adjustment at nighttime, the growth rate is lower than control. The temperature at daytime was same in both treatments, and thus we assumed that the growth rate at daytime is similar. Even though there is no significant difference in cell volume at 9 DAH (Fig. 1F), the growth rate of HNT treatment is about 75.8% of control (Fig. 5B). HNT treatment also accelerated grain development, and then the inner endosperm cells almost stopped to grow after 15 DAH.

### Chalky formation under HNT conditions

We have previously reported that osmotic adjustment is involved in ring-shaped chalky formation under 24 h foehn-induced dry wind conditions^8, 9, 32^. There is growing evidence that preservation of numerous vacuolar structures sustained among amyloplasts in the osmotically adjusted cells leads to chalky formation under dry wind conditions^32, 33^ and HDT conditions^16^. Based on the data, it is reasonably interpreted that shoot water deficit in this work might have progressed gradually as a ‘reversible acclimation process’. It has been noted that HNT-treated Koshihikari kernels exhibited chalkiness in outer endosperms along the basal side and dorsal vasculatures (see “Results” section, Fig. 1B). In rice endosperms, all the cells should have taken over same genomes from parents through double-fertilization. It should be emphasized that various chalkiness and/or reduction in cell size often coexist in the same endosperms, largely depending on the extent/duration of stress conditions, as addressed above. Therefore, these chalky phenotypes might refer to the consequence of various heat acclimations tightly coordinated with embryo growth. A possible interaction between water stored in chalky area and the promotion of embryo development has been suggested under HDT conditions^16^. In this view, there may be certain mechanism(s) for exchanging molecular signal(s) between embryo and endosperm.

### The impact of endosperm cell expansion under HNT conditions

It has been accepted that HNT decreases rice grain weight^2, 4, 34^, simultaneously causing chalky formation as described above (Fig. 1). Regarding the exact contributions of cell size reduction and chalky formation to the total grain weight loss, any quantitative analysis has not been provided yet. Since whole volume of control perfect rice and HNT-treated chalky rice was 15.63 and 14.87 mm^3^, respectively (Table 1), and the kernel weight was 20.78 and 19.64 mg, respectively, giving 1.13 mg difference as the total weight loss under HNT conditions; therefore, density of the perfect rice and HNT-treated chalky rice (basal-white and white-back rice) can be estimated as 1.33 and 1.32 mg mm^−3^, respectively. Embryo dry weight of the perfect rice and chalky rice of total kernel dry weight was negligibly small (both less than 4%). Assuming that grain density of control perfect rice and HNT-treated perfect rice is same, grain weight of HNT-treated perfect rice was estimated to be 19.77 (= 1.33 × 14.87) mg, and hence weight loss from chalky formation was calculated to be 0.12 (= 19.77–19.64) mg. And then, the contribution percentage of chalky formation to the reduction in kernel weight could be determined as 11.02(= 0.12/[20.78–19.64] × 100)%, and the rest 88.98 (= 100–11.02)% would be due to the reduction in cell expansion. Therefore, inhibition of cell expansion accounts for the kernel weight loss. Even if air spaces in chalky cells were filled with materials (mostly starch in the zone), the contribution percentage would decline. This estimation is quite reasonable, when considering that the area threshold above which chalkiness appears ranges between 10.3 and 25.1%^16^.

## Conclusion

In conclusion, plants were subjected to the moderate shoot water deficit at nighttime through an increase in VPD, and a partial inhibition of cell expansion was caused by osmotic adjustment. The observed size reduction in the growing cells was shown to occur as the consequence of osmotic adjustment. HNT-induced cellular responses were monitored by using picoPPESI-MS in intact plants at real time under controlled environments; it was found that these responses were dynamic in osmotically adjusted cells, accompanied with modification of hydraulic properties and wall and starch biosyntheses. Furthermore, the increase of relative concentration of several metabolites observed here strongly supports our hypothesis. Hence, we propose that the observed cell size reduction in inner endosperms would be attributed to osmotic adjustment at mild shoot water deficit caused by increasing VPD at night.

## Materials and methods

### Plant materials

A growth-chamber experiment was conducted in Kyushu Okinawa Agricultural Research Center, Chikugo, Japan in 2018. The experiment was laid out in a completely randomized design with two treatments (HNT and control) and at least three biological replcations for a total of 24 experimental pots. Two week-old *Oryza sativa* L. cv. ‘Koshihikari’ seedlings (ten seedlings per pot) were transplanted into plastic pots (3.82 L, diameter 0.16 m, and height 0.2 m) containing a lowland paddy soil (Typic Endoaquepts). They were grown by removing the tillers periodically to restrict each plant to its main culm to minimize sample-to-sample variation and cultivated in a cycle of day/night air temperatures of 28 °C (13 h, 5:50–18:50)/22 °C (11 h, 18:50–5:50) at 70/80% relative humidity (RH) and 750 μmol photons m^−2^ s^−1^ photosynthetically active radiation set at the plant canopy throughout development. At 5 DAH daytime, approximately half of the plants were transferred to another growth chamber set at 28 °C and 70% relative humidity/34 °C and 80% RH (day/night) and 750 μmol m^−2^ s^−1^ photosynthetically active radiation with the same photoperiod to be treated at high temperature for 10 days (referred to as ‘HNT treatment’). HNT treatment was initiated from 5 DAH nighttime (18:50). Other potted plants were kept in the same chamber as control (28/22 °C). At 15 DAH, the potted plants treated under HNT conditions were transferred to control chamber to grow until the mature stage (40 DAH).

### Whole-plant ^13^C distribution analyses

The plants were labeled with ^13^CO_2_ at 8 DAH (at the 3rd day after initiation of high night treatment) for the ^13^C distribution analysis described previously^9, 35^. The flag leaf was exposed to ^13^CO_2_. ^13^CO_2_ was applied by gently enclosing the leaf inside a 1.27-L polyester gas sampling bag (45 μm thickness, Analytic-Barrier, Ohmi Odor Air Services Inc., Tokyo, Japan) with a plastic container containing 0.5 g Ba^13^CO_3_ (99 atom % ^13^C) and ^13^CO_2_ was then generated by injecting 2 mL lactic acid from the outside of the bag. The leaf was allowed to assimilate ^13^C under the light conditions provided in the growth chamber for 30 min between 1100 and 1130 h. Plant parts were similarly harvested and separated into six components: flag leaf blade, flag leaf sheath, uppermost internode, whole panicle, superior kernels (used in the following cell metabolomics and turgor measurement), and other organs. The six tissue components were freeze-dried and the other organs were oven-dried with forced air, after which they were ground to a fine powder. For each tissue, approximately 1.0 mg well-mixed powder was used to determine total carbon and the isotopic ratio of ^12^C:^13^C using an element analyzer/isotopic ratio MS (Integra CN, Sercon, UK). Whole-plant ^13^C abundance was estimated according to Mohapatra et al.^35^.

### In situ intact cell metabolomics and turgor assay

We have used picoPPESI-MS^14^ to conduct in situ intact cell metabolomics in the inner endosperm under controlled environments, as described previously^16, 17^. The analytical method was performed in the expanding inner endosperm cells of the superior kernels, attached to the primary and secondary pedicels on the first to third primary rachis branches, counted from the top of the panicle (see Fig. 2A). And, grain score was monitored as described previously^9^. The system was composed of picoPPESI-MS and two cellular measurement rooms attached to growth chambers individually. When the kernel score reached to 0.8^9^ at 9 DAH, potted plants were placed at the center of a vibration-free table in the room. A part of the hull in the attached kernels was quickly and gently removed under humid conditions. And then, a biopsy punch was used to remove 0.031 cm^2^ of pericarp tissue in the lateral side of the kernel prior to the tip insertion to minimize the possible contamination effect from the pericarp cell layers. The kernel was gently fixed on the sample holder using tape and magnets. The microcapillary tip filled with 0.01% (v/v) ionic liquid/silicone oil mixture^14^ was impaled into the target inner endosperm cells (typically between 350 and 450 μm below nucellar-epidermis at the stage) with the aid of a motorized piezomanipulator. Cellular fluid was collected by depressurizing in the microcapillary, and the probe tip mounted on the 3D move/rotation micro-manipulator was immediately oriented toward the orifice of an Orbitrap mass spectrometer (Q-Exactive, Thermo Fisher Scientific Inc., MA, US) was electrified with − 4 kV using a high voltage generator (AKTB-05k1PN/S, Touwa Keisoku Corp., Tokyo, Japan). The MS scan was performed in negative ion mode in duplicate with the instrumental settings of 200 ms as maximum injection time, inlet ion transfer tube temperature of 250 °C, and resolution of 35,000. The intensity threshold has been set to be 1000 in this study. All the signals with less than the threshold had been rejected prior to the analysis. When the target cells were successfully impaled to collect picolitre cellular fluid without tip plugging, the entire process of picoPPESI-MS analysis on the cells was completed within few minutes. All manipulations were conducted under a digital microscope (KH-8700, HIROX Co. Ltd., Tokyo, Japan). Reported mass spectra are representative of the same experiments from 9 to 14 kernels from at least three independent plants in each treatment. Additionally, in situ cell turgor assay in the expanding endosperm located at the same lateral zone in the kernels was independently determined under humid conditions without removing the pericarp, as described previously^8^. Cell turgor values reported here represent averages of 12–15 cells from 5 to 8 kernels from at least 3 independent plants.

### Metabolite identification

The list of monoisotopic exact *m/z* values for all the peaks on acquired mass spectra were extracted using “Qual Browser” application in the Thermo Xcalibur software (Thermo Fisher Scientific Inc., MA, USA). According to the previous work^14^, the metabolites were identified at less than 5 ppm differences from the theoretical masses of candidate metabolites in an on-line metabolomics database, Metlin (http://metlin.scripps.edu/index.php). Endosperm tissues collected at the same stage were ground, and the extracts were centrifuged for 15 min at 4240×*g* at 4 °C, and the supernatant (crude tissue extract) was used for the MS/MS analysis. By using an Orbitrap MS (Orbitrap Elite, Thermo Fisher Scientific Inc., MA, the US) coupled with the picoPPESI system, collision-induced dissociation (CID) tandem MS analysis was performed for PI(34:2), allowing the MS/MS identification in the picolitre supernatant (i.e. crude tissue extract). The MS scan was performed in negative ion mode with the same settings as described above, except that the resolution was 120,000. Some of the metabolites were identified based on the results obtained by MS/MS analysis in our previous studies^15, 16^. All the standard chemicals and organic solvents used in the experiments were LC/MS grade purchased from Wako Pure Chemical Industries, Ltd. (Osaka, Japan). Ultrapure water of 18.2 MΩ cm^−1^ was used throughout the experiment.

### Plant water relations

Panicle water potentials at night were determined using a pressure-chamber technique (Pump*-*up model*,* PMS Instruments, OR, USA). For half of the samples, the tissue-averaged kernel water potential of 9 DAH spikelets attached to the same position, where the on-site cell metabolomics was conducted, was determined at nighttime (04:30 h to 05:30 h) with isopiestic psychrometers^36^ after determination of PWP according to the previous studies^8^. A linear regression was obtained from PWP by pressure-chamber and kernel water potential by isopiestic psychrometers. The reported kernel water potential was calculated by the linear regression.

### Microscopy

Microscopic observation was conducted according to Sato et al.^37, 38^ and Hatakeyama et al.^32^. Transverse segments (1–2 mm thick) from the middle of the kernel at 9, 15, and 35 DAH were fixed with 4% (w/v) paraformaldehyde in 100 mM sodium phosphate (pH 7.2) for 3 h at room temperature and then washed in 100 mM phosphate buffer (pH 7.2). Fixed tissues were dehydrated through an ethanol series, and embedded in LR White resin in the ‘hard’ formulation (London Resin, Hampshire, UK) by 2-days polymerizing at 60 °C. Semi-thin sections (appropriately 900 nm) for light microscopy were stained with 0.1% (w/v) Coomassie Brilliant Blue for 1 h followed by potassium iodide for 1 min, and ultra-thin sections (approximately 80–100 nm) for electron microscopy were stained with lead citrate. After the staining, ultra-thin sections were observed with a transmission electron microscope (TEM; JEM-1010, JEOL Ltd., Tokyo, Japan). Sections were cut with an ultramicrotome (Sorvall MT-5000, DuPont, Newtown, CT, USA) using a diamond knife. For the endosperm cell area analysis, the outline of inner endosperm cells was traced by using ImageJ software (National Institutes of Health, Bethesda, MD, USA). The number of cells in endosperm cross-sections along lateral sides from central point of the kernels in each treatment was counted.

### Kernel quality, weight, and dimensions

Superior kernels attached to the same position (top 3 branches, see Fig. 2A) of a panicle in each treatment were harvested. Grain length (2*r*_*1*_), width (2*r*_*2*_) and thickness (2*r*_*3*_) were measured by digital caliper (GDCS-300, Niigataseiki Co., Ltd., Niigata, Japan). Grain volume was calculated by V = 4/3π *r*_*1*_*r*_*2*_*r*_*3*_. The dry weight of kernel samples in each treatment was determined, as described previously^8^. The final grain weight was reported with 15% moisture content in each treatment. Reported grain weight represents averages of 5–6 independent plants (n = 5–6), and 8 superior grains (brown rice) were pooled in each plant (panicle). Grain dimension represents averages of 25 kernels collected from 5 independent plants (n = 25).

### Statistical analysis

Analysis of all data was performed using Student’s *t* test in JMP (version 12.1.0; SAS Institute Inc., Cary, NC, USA).

## Supplementary Information

Supplementary Information.

## Data Availability

The datasets generated during and/or analyzed during the current study are available from the corresponding author on reasonable request.
