# Supplementary material for: Endosperm cell size reduction caused by osmotic adjustment during nighttime warming in rice
Source: Sci Rep. 2021 Feb 24;11:4447. doi: 10.1038/s41598-021-83870-1 (PMC7904791; doi:10.1038/s41598-021-83870-1)
Supplement: Supplementary file 1 — Supplementary Information. [file 41598_2021_83870_MOESM1_ESM.pdf]

Supplementary Information

Type of articles: Research paper

Title: Endosperm Cell Size Reduction Caused by Osmotic Adjustment during Nighttime Warming in Rice

Authors: Hiroshi Wada[1, 2, 3†,*], Fang-Yu Chang[3,4, †], Yuto Hatakeyama[1, 2], Rosa Erra-Balsells[5], Takuya Araki[2,3], Hiroshi Nakano[1] and Hiroshi Nonami[2]

Affiliations

[1]Kyushu Okinawa Agricultural Research Center, National Agriculture and Food Research Organization, Chikugo, Fukuoka, Japan

[2]Graduate School of Agriculture, Ehime University, Matsuyama, Ehime, Japan

[3]The United Graduate School of Agricultural Sciences, Ehime University, Matsuyama, Ehime, Japan

[4]Kaohsiung District Agricultural Research and Extension Station, Council of Agriculture, Executive Yuan, Pingtung, Taiwan

[5]Department of Organic Chemistry and CIHIDECAR (CONICET), University of Buenos Aires, Buenos Aires, Argentina

[†]These authors contributed equally to this work.

*Corresponding author: Hiroshi Wada (ORCID ID 0000-0003-0510-5744; Phone +81-89-946-9824; Fax +81-89-946-9867; Email: hwada@agr.ehime-u.ac.jp)

The present address: Graduate School of Agriculture, Ehime University, 3-5-7 Tarumi, Matsuyama, Ehime, 790-8566, Japan

ORCID ID; Fang-Yu Chang (0000-0002-2103-0853), Yuto Hatakeyama (0000-0003-0526-4091), Rosa Erra-Balsells (0000-0003-0169-0173), Takuya Araki (0000-0001-5288-8054), Hiroshi Nakano (0000-0002-0564-2550), and Hiroshi Nonami (0000-0002-4336-9049)

E-mail Fang-Yu Chang: fychang@mail.kdais.gov.tw, Yuto Hatakeyama: hatakey@agr.ehime-u.ac.jp, Rosa Erra-Balsells: erra@qo.fcen.uba.ar, Takuya Araki: araki@agr.ehime-u.ac.jp, and Hiroshi Nakano: nakanohr@affrc.go.jp, and Hiroshi Nonami: nonami@agr.ehime-u.ac.jp

Table S1. List of the metabolites detected by using picoPPESI-MS in inner endosperm cells in each treatment.

Category	Metabolites	Ion molecule formula	Ion type detected [M=molecule]	Theoretical *m/z*[a]	Relative abundance (%)[b]		*P* value[c]	Relative abundance (%)[b]		*P* value[c]	Frequency of detection[e]				MS/MS shown in
					Control day	HN day		Control night	HN night		Control day	HN day	Control night	HN night	
					n=13[d]	n=14[d]		n=9[d]	n=9[d]		n=13[d]	n=14[d]	n=9[d]	n=9[d]	
Carbohydrate	Hex	C6H12O6	$[M-H]^-$	179.0561	2.47	2.21	0.52	3.76	2.43	*	++++	++++	++++	++++	Figure S4-12 in Wada et al. (2019)
	Hex	C6H12O6	$[M+Cl]^-$	215.0328	4.33	3.76	0.78	5.71	1.28	0.08	++++	++++	++++	++++	Figure S4-15 in Wada et al. (2019)
	HexP	C6H13O9P	$[M-H]^-$	259.0224	3.53	3.94	0.59	6.53	10.61	0.24	++++	++++	++++	++++	Figure S4-13 in Wada et al. (2019)
	Hex$_2$	C12H22O11	$[M-H]^-$	341.1089	12.85	8.62	0.10	26.83	14.52	0.12	++++	++++	++++	++++	Figure S4-14 in Wada et al. (2019)
	Hex$_2$	C12H22O11	$[M+Cl]^-$	377.0856	7.23	2.65	0.29	4.69	2.13	0.10	++++	++++	++++	++++	Figure S4-16 in Wada et al. (2019)
	Hex$_3$	C18H32O16	$[M-H]^-$	503.1618	0.02	0.05	0.12	0.07	0.10	0.53	++	++++	++++	++++	
	Hex$_3$	C18H32O16	$[M-H]^-$	539.1384	0.01	ND	0.13	ND	ND	-	++	-	-	-	
	Hex$_4$	C24H42O21	$[M-H]^-$	665.2146	0.00	0.00	0.69	0.01	0.02	0.33	+	+	+	++	
	Hex$_4$	C24H42O21	$[M-H]^-$	701.1913	ND	ND	-	ND	ND	-	-	-	-	-	
	Hex$_5$	C30H52O26	$[M-H]^-$	827.2674	ND	ND	-	0.00	ND	0.35	-	-	+	-	
	Hex$_5$	C30H52O26	$[M+Cl]^-$	863.2441	ND	ND	-	ND	ND	-	-	-	-	-	
	Pentose	C5H10O5	$[M-H]^-$	149.0455	0.09	0.14	0.15	0.16	0.13	0.18	++++	++++	++++	++++	
	Pentose	C5H10O5	$[M+Cl]^-$	185.0222	0.01	0.02	0.46	0.01	0.03	*	++	+++	+	++++	
	Pentose$_2$	C10H18O9	$[M-H]^-$	281.0878	0.02	0.02	0.76	0.01	0.00	0.58	+++	+++	++	+	
	Pentose$_2$	C10H18O9	$[M+Cl]^-$	317.0645	ND	ND	-	ND	ND	-	-	-	-	-	
Acids	Phosphoric acid	H3PO4	$[M-H]^-$	96.9696	8.02	17.14	0.15	7.75	26.20	0.10	++++	++++	++++	++++	
	3-Phosphoglyceric acid	C3H7O7P	$[M-H]^-$	184.9857	1.19	1.05	0.60	1.67	1.90	0.66	++++	++++	++++	++++	
	Phosphoenolpyruvic acid	C3H5O6P	$[M-H]^-$	166.9751	0.43	0.31	0.17	0.54	0.58	0.74	++++	++++	++++	++++	
	Pyruvic acid	C3H4O3	$[M-H]^-$	87.0088	0.94	0.82	0.64	0.70	1.71	*	++++	++++	++++	++++	
	Citric acid	C6H8O7	$[M-H]^-$	191.0197	2.38	6.42	0.32	2.81	5.56	0.07	++++	++++	++++	++++	
	α-Ketoglutaric acid	C5H6O5	$[M-H]^-$	145.0142	1.56	1.67	0.79	0.93	1.39	0.12	++++	++++	++++	++++	
	Fumaric acid	C4H4O4	$[M-H]^-$	115.0037	2.08	2.49	**	2.16	2.01	0.40	++++	++++	++++	++++	
	Succinic acid	C4H6O4	$[M-H]^-$	117.0193	15.71	10.37	*	11.31	10.04	0.49	++++	++++	++++	++++	
	Malic acid	C4H6O5	$[M-H]^-$	133.0142	100.00	100.00	-	100.00	89.15	0.15	++++	++++	++++	++++	Fig. S4-9 in Wada et al. (2019)
	Oxaloacetic acid	C4H4O5	$[M-H]^-$	130.9986	ND	0.01	0.25	ND	0.02	*	-	++	-	+++	
	Quinic acid	C7H12O6	$[M-H]^-$	191.0561	6.17	9.32	*	7.71	8.61	0.54	++++	++++	++++	++++	
	Shikimic acid	C7H10O5	$[M-H]^-$	173.0455	0.17	0.39	0.15	0.37	0.35	0.90	++++	++++	++++	++++	

(Continued from previous page)

Category	Metabolites	Ion molecule formula	Ion type detected [M=molecule]	Theoretical *m/z*[a]	Relative abundance (%)[b]		*P* value[c]	Relative abundance (%)[b]		*P* value[c]	Frequency of detection[e]				MS/MS shown in
					Control day	HN day		Control night	HN night		Control day	HN day	Control night	HN night	
					n=13[d]	n=14[d]		n=9[d]	n=9[d]		n=13[d]	n=14[d]	n=9[d]	n=9[d]	
Amino acids	Glycine	C2H5NO2	[M-H]$^-$	74.0248	0.11	0.14	0.47	0.12	0.22	0.07	++++	++++	++++	++++	
	Alanine	C3H7NO2	[M-H]$^-$	88.0404	3.58	2.91	0.43	3.81	7.09	0.10	++++	++++	++++	++++	
	Serine	C3H7NO3	[M-H]$^-$	104.0353	1.98	2.25	0.63	1.75	2.97	*	++++	++++	++++	++++	
	Serine	C3H7NO3	[M+Cl]$^-$	140.0120	0.12	0.19	0.16	0.16	0.50	0.10	++++	++++	++++	++++	
	Proline	C5H9NO2	[M-H]$^-$	114.0561	0.10	0.24	*	0.15	0.42	*	++++	++++	++++	++++	Fig. S4-2 in Wada et al. (2019)
	Valine	C5H11NO2	[M-H]$^-$	116.0717	0.43	0.35	0.23	0.52	0.76	0.14	++++	++++	++++	++++	
	Threonine	C4H9NO3	[M-H]$^-$	118.0510	0.80	0.71	0.46	0.71	0.79	0.59	++++	++++	++++	++++	
	Cysteine	C3H7NO2S	[M-H]$^-$	120.0125	0.20	0.27	0.41	0.26	0.30	0.71	++++	++++	++++	++++	
	Leucine, Isoleucine	C6H13NO2	[M-H]$^-$	130.0874	0.36	0.23	*	0.48	0.47	0.95	++++	++++	++++	++++	
	Asparagine	C4H8N2O3	[M-H]$^-$	131.0462	0.25	0.23	0.54	0.44	0.38	0.62	++++	++++	++++	++++	Fig. S4-3 in Wada et al. (2019)
	Aspartic acid	C4H7NO4	[M-H]$^-$	132.0302	6.27	25.90	**	7.79	10.33	0.34	++++	++++	++++	++++	Fig. S4-4 in Wada et al. (2019)
	Glutamine	C5H10N2O3	[M-H]$^-$	145.0619	1.65	2.79	*	8.47	2.24	***	++++	++++	++++	++++	Fig. S4-5 in Wada et al. (2019)
	Lysine	C6H14N2O2	[M-H]$^-$	145.0983	0.08	0.04	0.20	0.11	0.38	0.15	++++	++++	++++	++++	
	Glutamic acid	C5H9NO4	[M-H]$^-$	146.0459	45.04	44.28	0.90	47.88	55.80	0.45	++++	++++	++++	++++	Fig. S4-6 in Wada et al. (2019)
	Methionine	C5H11NO2S	[M-H]$^-$	148.0438	0.03	0.05	0.28	0.07	0.07	0.97	+++	+++	++++	++++	Fig. S4-7 in Wada et al. (2019)
	Histidine	C6H9N3O2	[M-H]$^-$	154.0622	0.32	0.08	***	0.17	0.15	0.50	++++	++++	++++	++++	
	Phenylalanine	C9H11NO2	[M-H]$^-$	164.0717	0.77	0.82	0.70	0.35	1.40	***	++++	++++	++++	++++	Fig. S4-8 in Wada et al. (2019)
	Arginine	C6H14N4O2	[M-H]$^-$	173.1044	0.03	0.01	0.10	0.08	0.33	0.19	++	++	++++	++++	
	Tyrosine	C9H11NO3	[M-H]$^-$	180.0666	0.54	0.44	0.27	0.57	1.01	0.21	++++	++++	++++	++++	
	Tryptophan	C11H12N2O2	[M-H]$^-$	203.0826	0.04	ND	**	0.01	0.01	0.65	+++	-	+	++	
	GABA	C4H9NO2	[M-H]$^-$	102.0561	0.63	1.01	0.20	0.56	1.26	0.10	++++	++++	++++	++++	Fig. S4-1 in Wada et al. (2019)
Proline synthesis related metabolites	Glutamate-gamma-semialdehyde	C5H9NO3	[M-H]$^-$	130.0510	0.03	0.04	0.51	0.04	0.08	0.07	+++	++++	++++	++++	
	Pyrroline-5-carboxylate (P5C)	C5H7NO2	[M-H]$^-$	112.0404	ND	0.00	0.30	ND	0.00	0.35	-	+	-	+	
Urea cycle	Citrulline	C6H13N3O3	[M-H]$^-$	174.0884	0.01	0.00	0.18	0.00	0.05	0.09	++	++	+	+++	
	Urea	CH4N2O	[M+Cl]$^-$	95.0018	0.00	ND	0.34	ND	ND	-	+	-	-	-	
	Ornithine	C5H12N2O2	[M-H]$^-$	131.0826	0.01	0.01	0.83	0.02	0.05	0.32	++	+	++	++	

(Continued from previous page)

Category	Metabolites	Ion molecule formula	Ion type detected [M=molecule]	Theoretical *m/z*[a]	Relative abundance (%)[b]		*P* value[c]	Relative abundance (%)[b]		*P* value[c]	Frequency of detection[e]				MS/MS shown in
					Control day	HN day		Control night	HN night		Control day	HN day	Control night	HN night	
					n=13[d]	n=14[d]		n=9[d]	n=9[d]		n=13[d]	n=14[d]	n=9[d]	n=9[d]	
Sulfur assimilation related metabolites	Sulfuric acid	H2SO4	$[M-H]^-$	96.9601	0.66	1.07	0.33	0.41	0.78	0.07	++++	++++	++++	++++	
	Sulfurous acid	H2SO3	$[M-H]^-$	80.9652	0.00	0.01	0.69	0.00	0.02	0.08	++++	+	+	+++	
	O-Acetylserine	C13H21N2O7PS	$[M-H]^-$	379.0734	0.03	0.09	0.14	0.03	0.62	0.07	++	+++	++	++++	
Plant hormones	Gibberellin A3	C19H22O6	$[M-H]^-$	345.1344	ND	0.00	0.34	ND	ND	-	-	+	-	-	
	Abscisic acid	C15H20O4	$[M-H]^-$	263.1289	ND	ND	-	ND	ND	-	-	-	-	-	
	3-Indoleacetic acid	C10H9NO2	$[M-H]^-$	174.0561	ND	0.01	0.08	ND	0.00	0.35	-	++	-	+	
	trans-Zeatin	C10H13N5O	$[M-H]^-$	218.1047	0.13	0.04	0.06	0.01	0.10	0.06	+++	++	++	++	
	Kinetin	C10H9N5O	$[M-H]^-$	214.0734	ND	ND	-	ND	ND	-	-	-	-	-	
	Salicylic acid	C7H6O3	$[M-H]^-$	137.0244	0.07	0.11	0.07	0.05	0.12	**	++++	++++	++++	++++	
	1-Aminocyclopropane-1-carboxylic acid (ACC)	C4H7NO2	$[M-H]^-$	100.0404	0.01	0.02	0.29	0.01	0.06	*	++	+++	++	++++	
Jasmonic acid related metabolite	Linoleic acid	C18H32O2	$[M-H]^-$	279.2330	0.03	0.02	0.64	0.08	0.05	0.54	++	++	++++	+++	
	Jasmone	C11H16O	$[M-H]^-$	163.1128	ND	0.01	0.09	0.00	0.02	0.09	-	+++	+	+++	
	Jasmone	C11H16O	$[M+Cl]^-$	199.0895	0.00	ND	0.34	ND	ND	-	+	-	-	-	
Polyamine degradation	N1-Acetylspermidine	C9H21N3O	$[M-H]^-$	186.1612	ND	ND	-	ND	ND	-	-	-	-	-	
	N1-Acetylspermine	C12H28N4O	$[M-H]^-$	243.2190	ND	ND	-	ND	ND	-	-	-	-	-	
	3-Aminopropanal	C3H7NO	$[M+Cl]^-$	108.0222	0.00	0.03	*	0.01	0.13	*	+	+++	++	++++	
	4-Methylaminobutanal	C5H11NO	$[M+Cl]^-$	136.0535	0.00	0.02	0.08	0.01	0.05	**	+	+++	++	++++	
	Thermospermine	C10H26N4	$[M+Cl]^-$	237.1851	ND	0.00	0.26	ND	ND	-	-	+	-	-	
Energy related metabolites	ATP	C10H16N5O13P3	$[M-H]^-$	505.9885	ND	ND	-	ND	ND	-	-	-	-	-	
	ADP	C10H15N5O10P2	$[M-H]^-$	426.0221	0.02	0.02	0.70	0.04	0.16	0.08	++	+++	+++	++++	
	AMP	C10H14N5O7P	$[M-H]^-$	346.0558	0.42	0.30	0.28	0.51	0.77	0.36	++++	++++	++++	++++	
	Pyrophosphate (PPi)	H4O7P2	$[M-H]^-$	176.9359	0.00	0.02	0.11	0.01	0.03	0.21	++	+++	++	+++	

(Continued from previous page)

Category	Metabolites	Ion molecule formula	Ion type detected [M=molecule]	Theoretical *m/z*[a]	Relative abundance (%) [b]		*P* value [c]	Relative abundance (%) [b]		*P* value [c]	Frequency of detection [e]				MS/MS shown in
					Control day	HN day		Control night	HN night		Control day	HN day	Control night	HN night	
					n=13[d]	n=14[d]		n=9[d]	n=9[d]		n=13[d]	n=14[d]	n=9[d]	n=9[d]	
Plant cell wall related metabolites	UDP-glucose	C15H24N2O17P2	$[M-H]^-$	565.0477	0.16	0.30	0.15	0.22	0.69	*	++++	++++	++++	++++	
	UDP-D-Xylose / UDP-L-arabinose	C14H22N2O16P2	$[M-H]^-$	535.0372	0.00	0.01	0.39	0.01	0.06	*	+	++	++	+++	
	UDP glucuronic acid	C15H22N2O18P2	$[M-H]^-$	579.0270	ND	0.00	0.34	ND	ND	-	-	+	-	-	
	D-Glucuronic acid	C6H10O7	$[M-H]^-$	193.0354	0.16	0.19	0.43	0.24	0.18	0.43	++++	++++	++++	++++	
	D-Glucuronic acid	C6H10O7	$[M+Cl]^-$	229.0121	0.36	0.41	0.42	0.54	0.93	0.15	++++	++++	++++	++++	
	α-L-Rhamnose	C6H12O5	$[M-H]^-$	163.0612	0.63	1.42	*	1.77	3.57	0.09	++++	++++	++++	++++	
	Kdo	C8H14O8	$[M-H]^-$	237.0616	0.20	0.22	0.40	0.23	0.31	0.12	++++	++++	++++	++++	
	Cinnamic acid	C9H8O2	$[M-H]^-$	147.0452	0.00	0.00	0.93	ND	0.00	0.35	+	+	-	+	
	p-Coumaric acid	C9H8O3	$[M-H]^-$	163.0401	0.01	0.01	0.73	ND	0.03	*	++	+++	-	+++	Fig. S3A.1 in Blokhina et al. (2019)
	Coniferin	C16H22O8	$[M-H]^-$	341.1242	ND	ND	-	ND	ND	-	-	-	-	-	
	Coniferin	C16H22O8	$[M+Cl]^-$	377.1009	ND	ND	-	ND	ND	-	-	-	-	-	
	p-Coumaryl alcohol 4-glucoside	C15H20O7	$[M-H]^-$	311.1136	ND	0.00	0.34	0.00	0.00	0.64	-	+	+	+	Fig. S3A.8 in Blokhina et al. (2019)
	p-Coumaryl alcohol 4-glucoside	C15H20O7	$[M+Cl]^-$	347.0903	ND	ND	-	ND	ND	-	-	-	-	-	
	Coniferyl alcohol	C10H12O3	$[M-H]^-$	179.0714	ND	0.00	0.34	ND	0.00	0.35	-	+	-	+	Fig. S3A.4 in Blokhina et al. (2019)
	p-Coumaryl alcohol	C9H10O2	$[M-H]^-$	149.0608	0.00	0.00	0.22	0.00	0.02	0.18	+	++	+	++	Fig. S3A.5 in Blokhina et al. (2019)
	Sinapyl-alcohol	C11H14O4	$[M-H]^-$	209.0819	ND	ND	-	ND	0.00	0.35	-	-	-	+	
	Ferulic acid	C10H10O4	$[M-H]^-$	193.0506	ND	0.00	0.05	ND	ND	-	-	++	-	-	
Lipid peroxidation/ Cell fermentation	Malondialdehyde	C3H4O2	$[M-H]^-$	71.0139	0.46	0.41	0.41	0.41	0.43	0.83	++++	++++	++++	++++	
	13-Hydroxy octadecadienoic acid	C18H32O3	$[M-H]^-$	295.2279	0.00	0.00	0.75	ND	ND	-	+	+	-	-	
	4-Hydroxynonenal	C9H16O2	$[M-H]^-$	155.1078	0.00	0.03	0.09	0.01	0.05	**	+	+++	+++	++++	
	Lactic acid	C3H6O3	$[M-H]^-$	89.0244	2.85	2.79	0.94	3.34	3.77	0.68	++++	++++	++++	++++	
	Lactic acid	C3H6O3	$[M+Cl]^-$	125.0011	0.04	0.02	0.18	0.02	ND	*	+++	+++	++	-	

(Continued from previous page)

Category	Metabolites	Ion molecule formula	Ion type detected [M=molecule]	Theoretical *m/z*[a]	Relative abundance (%)[b]		*P* value [c]	Relative abundance (%)[b]		*P* value[c]	Frequency of detection[e]				MS/MS shown in
					Control day	HN day		Control night	HN night		Control day	HN day	Control night	HN night	
					n=13[d]	n=14[d]		n=9[d]	n=9[d]		n=13[d]	n=14[d]	n=9[d]	n=9[d]	
Cyanide detoxification	3-Cyano-L-alanine	C4H6N2O2	$[M-H]^-$	113.0357	0.00	0.00	*	ND	0.00	0.18	+	++	-	+	
	3-Cyano-L-alanine	C4H6N2O2	$[M+Cl]^-$	149.0123	ND	ND	-	ND	ND	-	-	-	-	-	
	2-Aminoacrylic acid	C3H5NO2	$[M-H]^-$	86.0248	0.00	0.02	0.07	0.00	0.05	**	++	+++	+	++++	
	2-Aminoacrylic acid	C3H5NO2	$[M+Cl]^-$	122.0014	ND	ND	-	0.00	0.00	0.81	-	-	+	+	
Ascorbate-glutathione cycle	Ascorbic acid	C6H8O6	$[M-H]^-$	175.0247	9.67	12.80	*	11.21	15.38	0.16	++++	++++	++++	++++	Fig. S4-11 in Wada et al. (2019)
	Ascorbic acid	C6H8O6	$[M+Cl]^-$	211.0015	0.17	0.16	0.87	0.23	1.99	0.31	++++	++++	++++	++++	
	Monodehydroascorbic acid	C6H7O6	$[M-H]^-$	174.0170	0.32	0.47	0.07	0.43	1.11	***	++++	++++	++++	++++	Fig. S4-10 in Wada et al. (2019)
	Monodehydroascorbic acid	C6H7O6	$[M+Cl]^-$	209.9937	ND	ND	-	ND	ND	-	-	-	-	-	
	Dehydroascorbic acid	C6H6O6	$[M-H]^-$	173.0092	0.10	0.19	0.06	0.09	0.31	**	++++	++++	++++	++++	
	Dehydroascorbic acid	C6H6O6	$[M+Cl]^-$	208.9858	0.01	0.03	0.23	0.00	0.11	*	+	++	+	++++	
	Glutathione	C10H17N3O6S	$[M-H]^-$	306.0765	1.94	1.83	0.84	2.15	3.93	*	++++	++++	++++	++++	Fig. S4-17 in Wada et al. (2019)
	Glutathione	C10H17N3O6S	$[M+Cl]^-$	342.0532	ND	ND	-	ND	ND	-	-	-	-	-	
	Oxidized glutathione (GSSG)	C20H32N6O12S2	$[M-H]^-$	611.1447	0.00	0.02	0.06	ND	0.07	0.07	+	++	-	++	
	Oxidized glutathione (GSSG)	C20H32N6O12S2	$[M+Cl]^-$	647.1214	ND	0.01	0.20	0.01	0.12	0.16	-	+	++	+++	
Other metabolites	Adenine	C5H5N5	$[M-H]^-$	134.0472	0.35	0.56	0.23	0.70	1.20	0.16	++++	++++	++++	++++	
	Glycerol	C3H8O3	$[M-H]^-$	91.0401	0.00	0.01	*	0.01	0.03	0.14	+	++	++	+++	
	Uridine diphosphate (UDP)	C9H14N2O12P2	$[M-H]^-$	402.9949	0.04	0.05	0.68	0.10	0.34	*	+++	++++	++++	++++	
	PE(16:0/18:3 (9Z,12Z,15Z))	C39H72NO8P	$[M-H]^-$	712.4922	ND	ND	-	0.00	ND	0.35	-	-	+	-	
	PI(16:018:2(9Z,12Z))	C43H79O13P	$[M-H]^-$	833.5186	0.79	0.60	0.51	1.20	0.37	*	++++	++++	++++	++++	Fig. S6

(Continued from previous page)

Category	Metabolites	Ion molecule formula	Ion type detected [M=cluster][f]	Theoretical *m/z*[a]	Relative abundance (%)[b]		*P* value [c]	Relative abundance (%)[b]		*P* value[c]	Frequency of detection[e]				MS/MS shown in
					Control day	HN day		Control night	HN night		Control day	HN day	Control night	HN night	
					n=13[d]	n=14[d]		n=9[d]	n=9[d]		n=13[d]	n=14[d]	n=9[d]	n=9[d]	
Cluster ions	Malic acid+Hex	C10H18O11	$[M-H]^-$	313.0776	0.30	0.86	*	0.94	2.82	0.08	++++	++++	++++	++++	
	Phosphoric acid+Hex	C6H15O10P	$[M-H]^-$	277.0330	0.60	0.64	0.81	5.90	0.50	0.34	++++	++++	++++	++++	
	Succinic acid+Hex	C10H18O10	$[M-H]^-$	297.0827	0.28	0.15	0.05	0.62	0.10	0.11	++++	++++	++++	++++	
	Proline+Hex	C11H21NO8	$[M-H]^-$	294.1194	0.01	0.04	0.12	0.01	0.02	0.29	++	+++	+++	+++	
	Cysteine+Hex	C9H19NO8S	$[M-H]^-$	300.0759	0.05	0.05	0.96	0.12	0.03	*	+++	+++	++++	++	
	Asparatic acid+Hex	C10H19NO10	$[M-H]^-$	312.0936	0.02	0.14	**	0.02	0.01	0.71	++	+++	++	++	
	Ascorbic acid+Hex	C12H20O12	$[M-H]^-$	355.0881	0.01	0.02	0.20	0.01	0.03	0.40	++	+++	++	++	
	Glutamic acid+Hex	C11H21NO10	$[M-H]^-$	326.1093	0.55	0.50	0.70	0.70	0.37	0.11	++++	++++	++++	++++	
	Glutathione+Hex	C16H29N3O9S	$[M-H]^-$	486.1399	ND	ND	-	ND	ND	-	-	-	-	-	
	Malic acid+Hex_2	C16H28O16	$[M-H]^-$	475.1305	2.36	1.41	0.13	2.66	1.80	0.42	++++	++++	++++	++++	
	Phosphoric acid+Hex_2	C12H25O15P	$[M-H]^-$	439.0859	4.47	3.43	0.18	5.42	5.18	0.91	++++	++++	++++	++++	
	Succinic acid+Hex_2	C16H28o15	$[M-H]^-$	459.1356	1.12	0.41	**	1.13	0.67	0.27	++++	++++	++++	++++	
	Ascorbic acid+Hex_2	C18H30O17	$[M-H]^-$	517.1410	0.10	0.10	0.94	0.14	0.23	0.51	++++	++++	++++	+++	
	Proline+Hex_2	C17H31NO13	$[M-H]^-$	456.1723	0.05	0.07	0.29	0.10	0.19	0.24	+++	++++	++++	++++	
	Glutamic acid+Hex_2	C17H31NO15	$[M-H]^-$	488.1622	4.28	2.82	0.12	5.13	4.55	0.78	++++	++++	++++	++++	
	Asparatic acid+Hex_2	C16H29NO15	$[M-H]^-$	474.1465	0.51	1.66	*	0.74	0.55	0.49	++++	++++	++++	++++	
	Cysteine+Hex_2	C15H29NO13S	$[M-H]^-$	462.1288	0.09	0.10	0.84	0.16	0.11	0.49	+++	+++	++++	+++	Fig. S4-18 in Wada et al. (2019)
	Alanine+Hex_2	C15H29NO13	$[M-H]^-$	430.1567	3.80	1.59	**	4.50	7.14	0.39	++++	++++	++++	++++	
	HexP+Hex_2	C18H35O20P	$[M-H]^-$	601.1387	0.62	0.55	0.78	1.04	0.98	0.88	++++	++++	++++	++++	
	Serine+Hex_2	C15H29NO14	$[M-H]^-$	446.1516	1.47	1.06	0.15	1.85	1.86	0.98	++++	++++	++++	++++	
	Glutathione+Hex_2	C22H39N3O17S	$[M-H]^-$	648.1928	0.05	0.02	0.37	0.04	0.08	0.43	+++	++	+++	++	
	$(Hex_2)_2$	C24H44O22	$[M-H]^-$	683.2251	1.48	0.81	0.08	2.40	0.93	0.06	++++	++++	++++	++++	
	$(Hex_2)_2$	C24H44O22	$[M+Cl]^-$	719.2018	0.18	0.02	0.29	0.11	0.03	0.31	+++	++	+++	+	

Table S1. List of the metabolites detected by using picoPPESI-MS in the inner endosperm cells in each treatment.

[a] All the theoretical values are quoted from Metlin (http://metlin.scripps.edu/index.php).

[b] Values were calculated as a percentage to the base peak. ND indicates not detected.

[c] Different letters indicate a significant difference (Student *t*-test, $P < 0.05$, 0.01 and 0.001 between treatments is indicated by*, **, and ***, respectively.)

[d] Biological replications (number of grains).

[e] Signal detected (+); Signal not detected (-). Frequencies of detection in 9-14 individual measurements are indicated as: ++++; 75%<x ≦100%, +++; 50%< x ≦ 75%, ++; 25%< x ≦ 50%, +; 0%< x ≦25%.

[f] Cluster: is a molecular aggregate stably formed in solution and in gas state because the intermolecular interaction among the molecular unities are quite strong (e.g., hydrogen bridge); its formation depends on the concentration of the molecular constituent in the analyzed solution; at higher concentrations the chance to obtain cluster signals in electrospray ionization mass spectrometry is higher. PicoPPESI mass spectrometry is an electrospray ionization mass spectrometry technique.

Figure S1

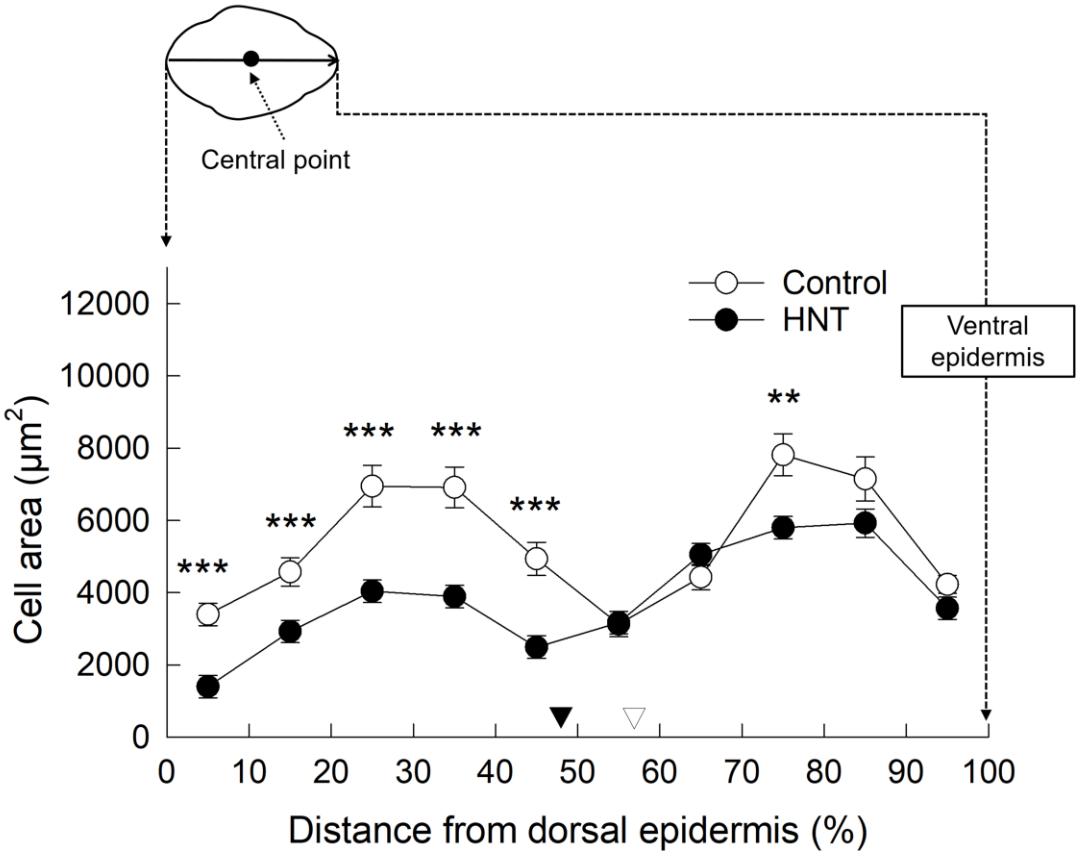

Figure S1. Endosperm cell area plotted against the distance (in %) from dorsal epidermis to ventral epidermis on the transverse sections of control (white circles) and HNT-treated (black circles) rice kernel at maturation (see inset). White and black triangles represent the position of central point of control and HNT treatment, respectively. Data are the mean ± SE of 32-80 individual cells collected in 4-5 kernels from three plants. *,** and *** show $p < 0.05$, 0.01, and 0.001 by *t*-test, respectively.

Figure S2

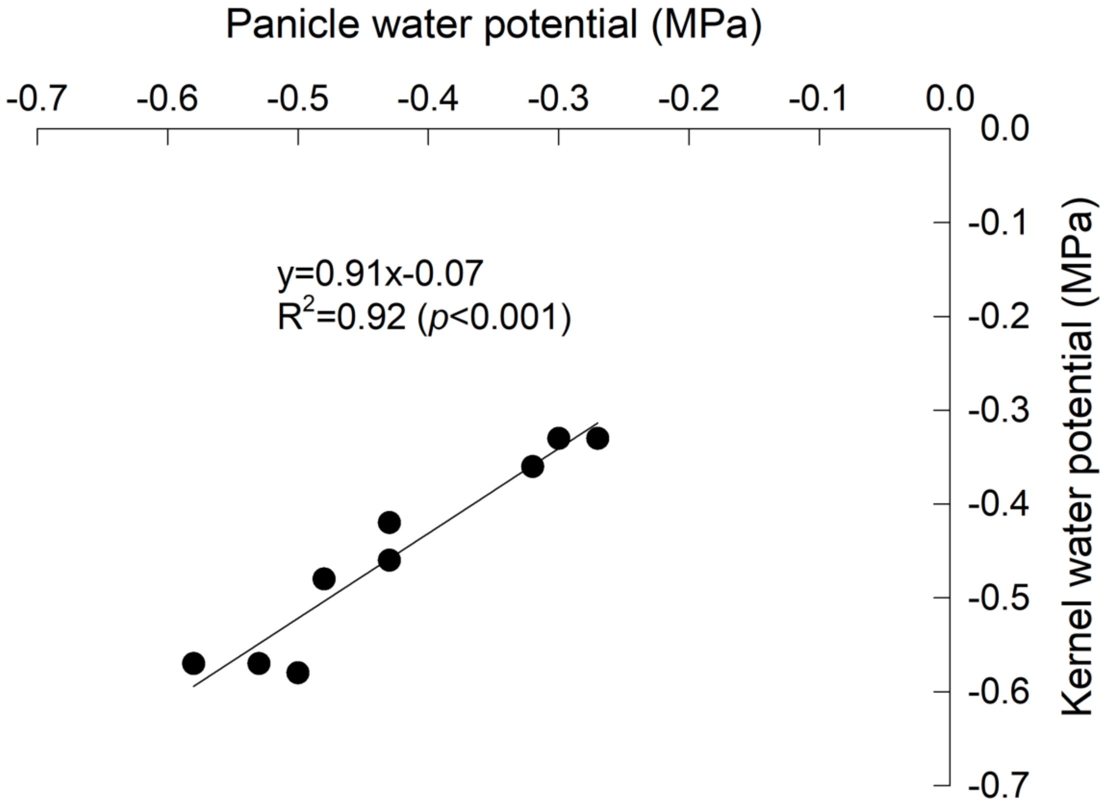

Figure S2. Kernel water potential measured with the isopiestic psychrometer as a function of panicle water potential measured with a pressure chamber. The solid line indicates a linear regression line.

Figure S3

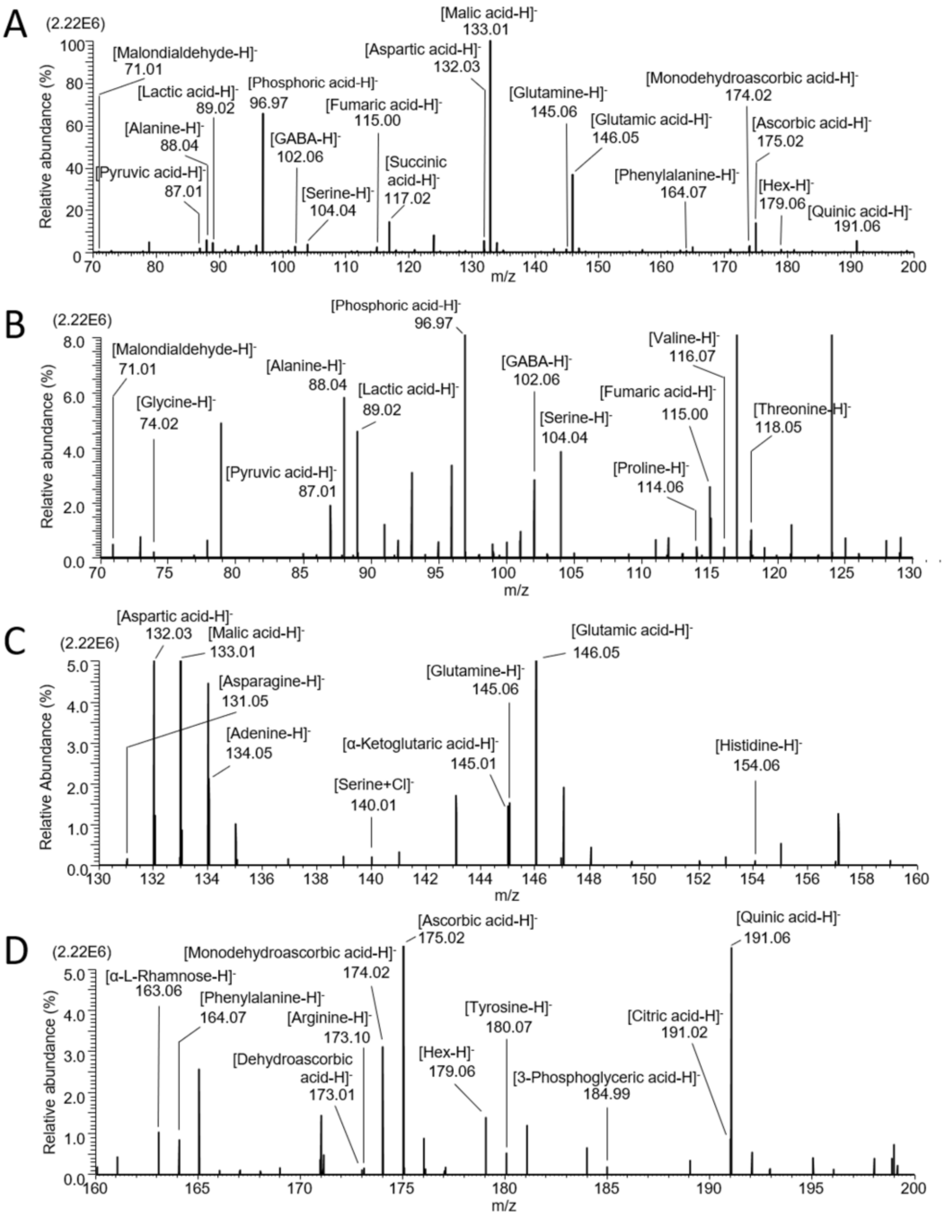

Figure S3. PicoPPESI mass spectra in negative ion mode obtained from the cells in HNT treatment at 9 DAH nighttime. The full mass spectrum is shown in **Fig. 3C**. Detail: range of *m/z* 70-200 (**A**), 70-130 (**B**), 130-160 (**C**), 160-200 (**D**).

Figure S4

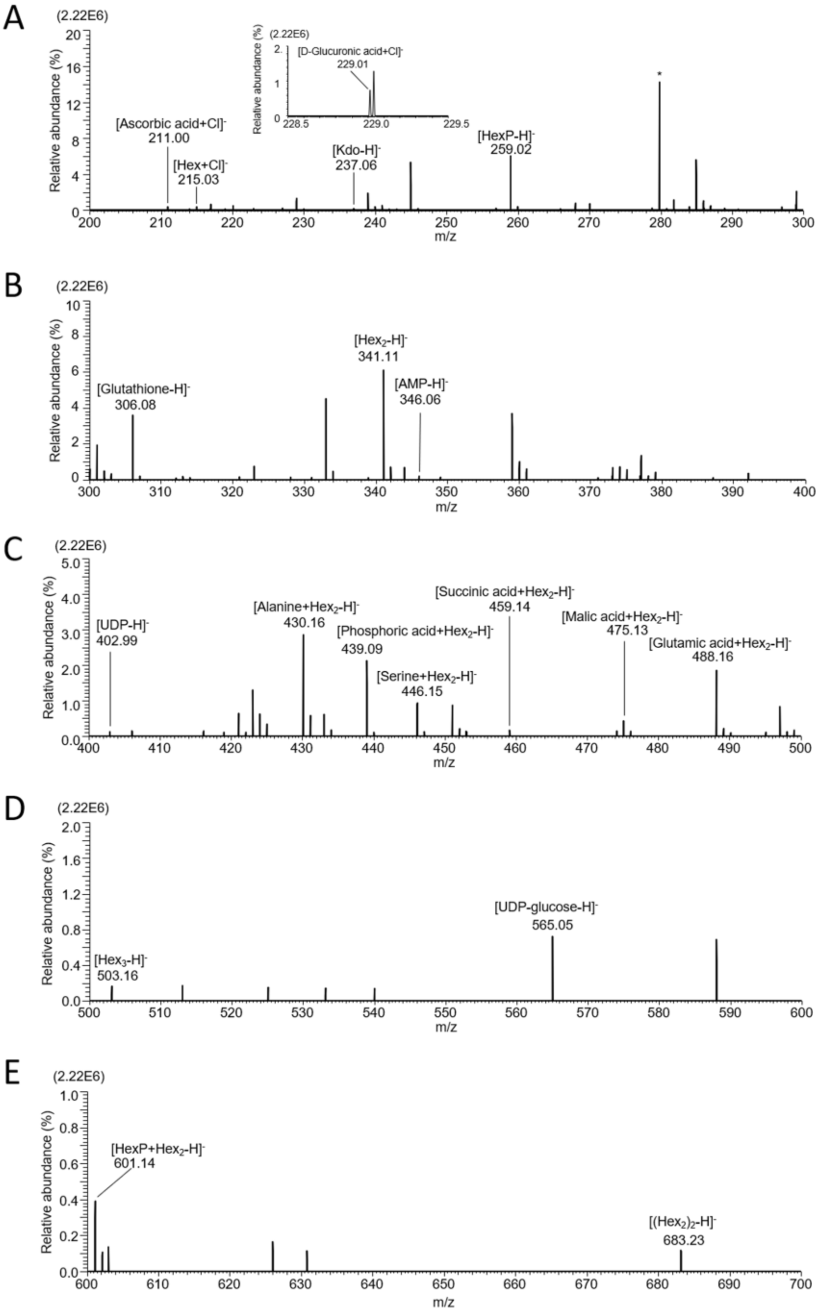

Figure S4. PicoPPESI mass spectra in negative ion mode obtained from the cells in HNT treatment at 9 DAH nighttime. The full mass spectrum is shown in **Fig. 3C**. Details: range of *m/z* 200-300 (**A**), 300-400 (**B**), 400-500 (**C**), 500-600 (**D**), 600-700 (**E**).

Figure S5

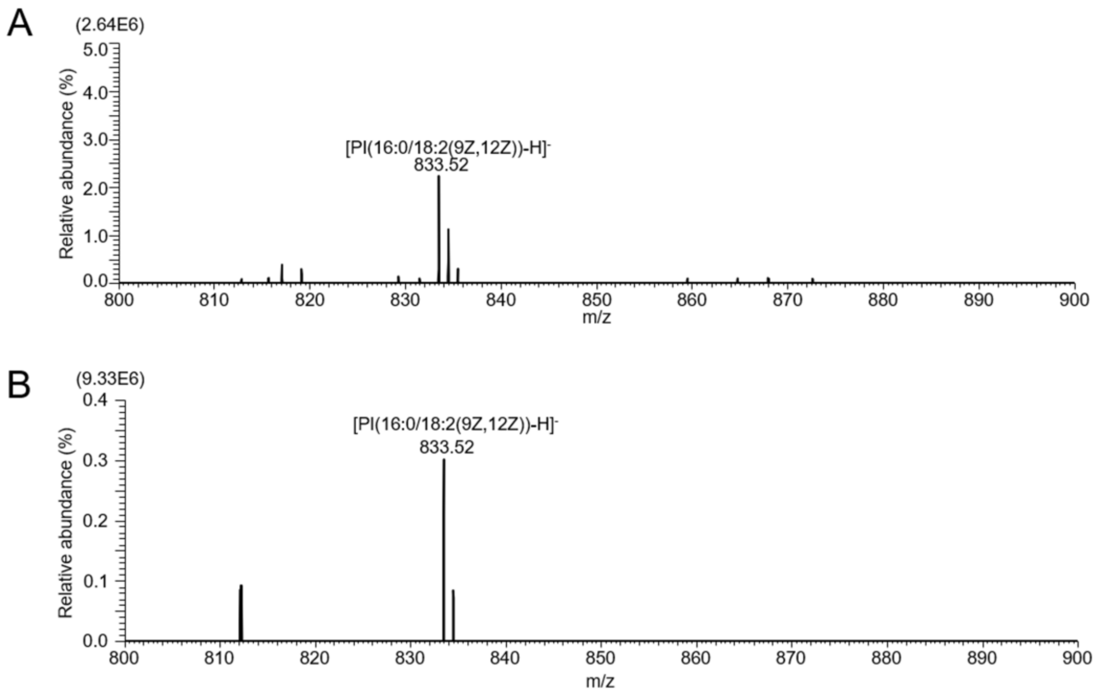

Figure S5. PicoPPESI mass spectra in negative ion mode obtained from the cells in control (**A**) and HNT treatment (**B**) at 9 DAH nighttime. The full mass spectrum is shown in **Fig. 3C**. Range of *m/z* 800-900. The MS/MS is shown in **Fig. S6**.

Figure S6

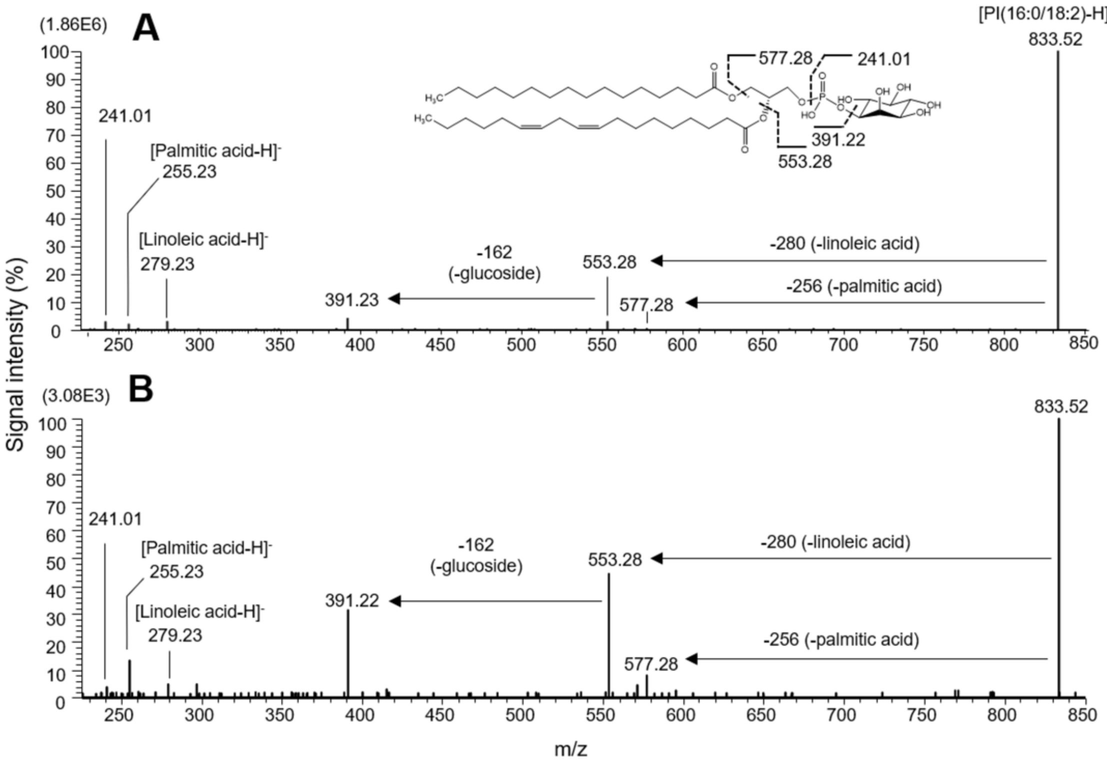

Figure S6. PicoPPESI-MS/MS spectra. **(A)** Standard L-α-phosphatidylinositol (PI 16:0/18:2) (precursor ion: [M-H]-, *m/z* 833.52) solution from *Glycine max* in negative ion mode. Prior to the analysis, the standard solution was diluted 100-fold (chloroform/methanol/distilled water, 70/27/3, v/v) (modified from **Fig.S2A** in Wada et al., 2020). (**B**) Putative PI (16:0/18:2) peak ([M-H]-, *m/z* 833.52) obtained from extract solution from rice endosperm tissue in negative ion mode. Precursor ion *m/z* 833.52; selector gate range *m/z* 833.52±0.5; normalized collision energy was set to be 30 %; CID fragmentation ions were detected in the Thermo Scientific Orbitrap Elite at a resolution setting of 120,000. According to the MS/MS spectrum, the precursor ion in **B** was identified as [PI (16:0/18:2)-H]-.
